# Cadmium promotes nonalcoholic fatty liver disease by inhibiting intercellular mitochondrial transfer

**DOI:** 10.1186/s11658-023-00498-x

**Published:** 2023-10-27

**Authors:** Jian Sun, Yan Chen, Tao Wang, Waseem Ali, Yonggang Ma, Yan Yuan, Jianhong Gu, Jianchun Bian, Zongping Liu, Hui Zou

**Affiliations:** 1 College of Veterinary Medicine, Yangzhou University, Yangzhou, China; 2 Jiangsu Co-Innovation Center for Prevention and Control of Important Animal Infectious Diseases and Zoonoses, Yangzhou, China

**Keywords:** Cadmium, Intercellular mitochondrial transfer, Tunneling nanotubes, Nonalcoholic fatty liver disease

## Abstract

Mitochondrial transfer regulates intercellular communication, and mitochondria regulate cell metabolism and cell survival. However, the role and mechanism of mitochondrial transfer in Cd-induced nonalcoholic fatty liver disease (NAFLD) are unclear. The present study shows that mitochondria can be transferred between hepatocytes via microtubule-dependent tunneling nanotubes. After Cd treatment, mitochondria exhibit perinuclear aggregation in hepatocytes and blocked intercellular mitochondrial transfer. The different movement directions of mitochondria depend on their interaction with different motor proteins. The results show that Cd destroys the mitochondria-kinesin interaction, thus inhibiting mitochondrial transfer. Moreover, Cd increases the interaction of P62 with Dynactin1, promotes negative mitochondrial transport, and increases intracellular lipid accumulation. Mitochondria and hepatocyte co-culture significantly reduced Cd damage to hepatocytes and lipid accumulation. Thus, Cd blocks intercellular mitochondrial transfer by disrupting the microtubule system, inhibiting mitochondrial positive transport, and promoting their negative transport, thereby promoting the development of NAFLD.

**Graphical Abstract:**

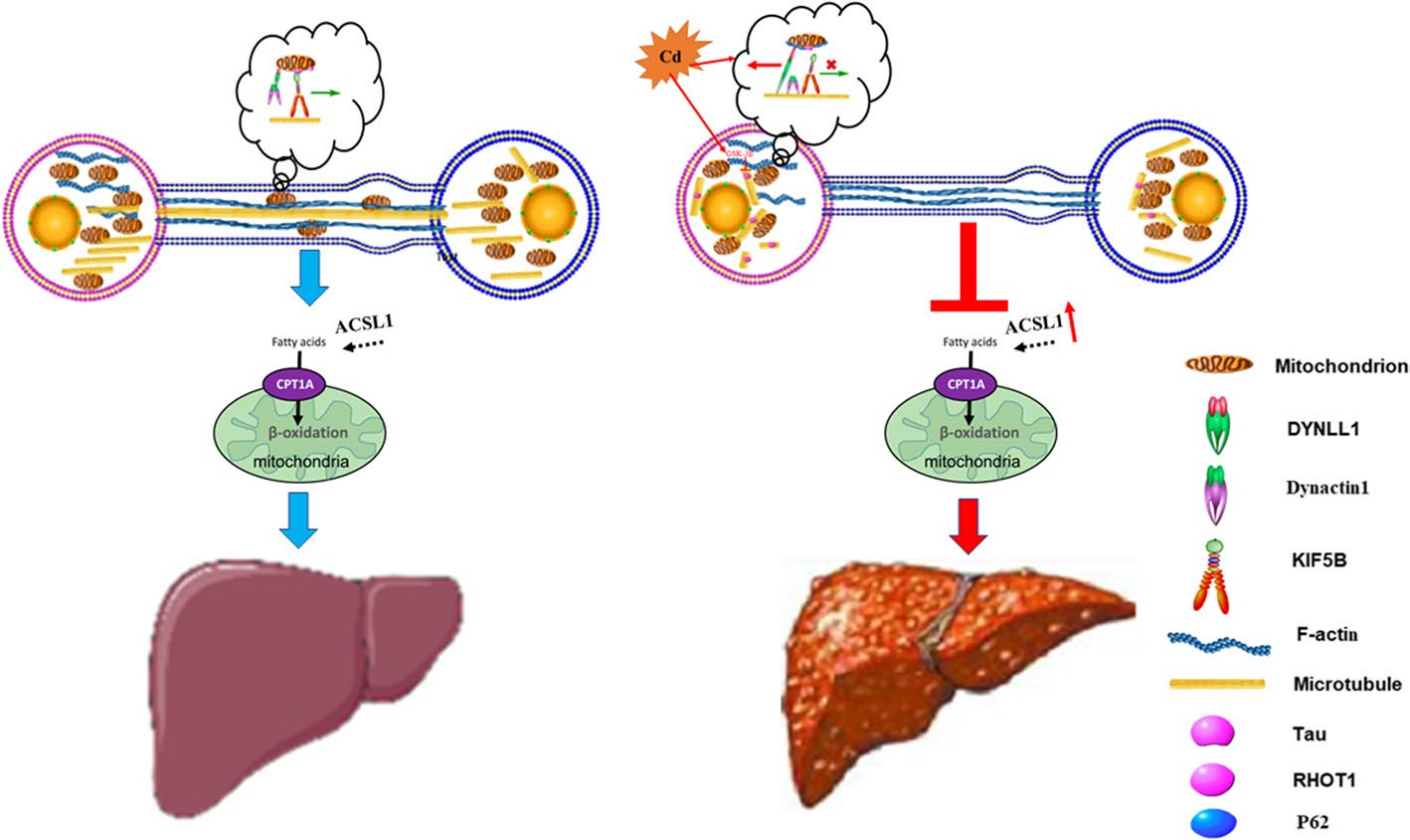

**Supplementary Information:**

The online version contains supplementary material available at 10.1186/s11658-023-00498-x.

## Highlights

1. Mitochondrial transfer between hepatocytes can be achieved through microtubule-dependent tunneling nanotubes
2. Cadmium inhibits the intercellular mitochondrial transfer
3. Inhibition of intercellular mitochondrial transfer mediates cadmium-induced nonalcoholic fatty liver disease.
4. Motor protein-dependent mitochondrial mobility is a target for cadmium to inhibit intercellular mitochondrial transfer

**Supplementary Information:**

The online version contains supplementary material available at 10.1186/s11658-023-00498-x.

## Introduction

Changes in people’s living habits and rhythms can exert pressure on the liver. Non-alcoholic fatty liver disease (NAFLD) has emerged as one of the most important chronic liver diseases, affecting more than 25% of the world’s population; however, its pathogenesis is not well understood [1]. In the 1990s, a model involving multiple pathogenic factors was proposed [2]. Epidemiological findings showed that cadmium (Cd) was associated with the development of NAFLD [3]. Cd is known for its long half-life, and once it enters the body, it can cause damage to many organs body and promote the development of a variety of diseases [4].

The liver relies on a large amount of energy to metabolize substances, and energy production depends on mitochondria [5, 6]. Mitochondria are highly active organelles within cells, and different structures of mitochondria and their cellular localizations also predict different fates [7, 8]. Motor proteins interact with mitochondria to drive mitochondrial movement along microtubules [9]. Previous studies have shown that mitochondria not only serve as the main site of cellular metabolism, but also regulate cell fate by interacting with other organelles [10, 11]. With the deepening of our understanding of cell-to-cell communication, we were surprised to find that mitochondria can also communicate across cells in different ways [12]. Tunneling nanotubes (TNTs) are cytoskeleton-dependent, long-distance channels between cells, which allow the passage of a variety of substances, such as proteins, nucleic acids, organelles, and even viruses and mycoplasmas. Nerve tissue, bone tissue, and adipose tissue have all been found to undergo mitochondria transport between cells via TNTs [13–15]. Stem cell-based cell therapy is one way to repair tissue damage, and research shows that stem cells can alleviate cellular damage by contributing their own mitochondria to damaged cells [16] Although TNT and mitochondrial transfer have been observed in multiple models, the key targets regulating TNT formation and mitochondrial transfer are unclear. Disruption of mitochondrial function is one of the predisposing causes of nonalcoholic fatty liver disease [17]; however, it is unclear whether intercellular mitochondrial transport is involved in Cd-induced NAFLD. In this study, we aimed to explore whether mitochondrial transport and the effects of Cd also exist in cells of the liver parenchyma, and the role and mechanism of mitochondrial transport in Cd-induced NAFLD.

## Experimental

### Chemicals and antibodies

The following antibodies and chemicals were used: AAV8-RHOT1 (83070-1) and a RHOT1OE plasmid (Genechem, Shanghai, China); vector pCMV-Mito-AT1.03, Carboxyfluorescein diacetate succinimidyl ester (CFDA-SE), Tubulin-Tracker-Green, Tubulin-Tracker-Red, an Oil Red O Staining Kit, and Hoechst 33258 (Beyotime Biotechnology, Shanghai, China); and Tetramethylrhodamine (TRITC) Phalloidin, fluorescein isothiocyanate (FITC) Phalloidin, Micro Mitochondrial Respiratory Chain Complex I, III, IV, and V activity Assay Kits, an Mitochondrial extraction kit, and a Ca^2+^-Mg^2+^-ATPase activity assay Kit (Solarbio, Beijing, China). Mito-tracker-Red was obtained from Thermo Fisher Scientific (Waltham, MA, USA). Antibodies against the following proteins were used: Translocase of outer mitochondrial membrane 20 (TOMM20) (#42406), carnitine palmitoyltransferase 1 (CPT1) (#97361), acyl-CoA synthetase long chain family member 1 (ACSL1) (#9189), glycogen synthase kinase 3 beta (GSK-3β) (#12456), Tau (#46687), β-actin (#4970), cytochrome C oxidase subunit 4 (COX IV) (#4850), and sequestosome 1 (P62) (#23214) (all Cell Signaling Technology, Danvers, MA, USA). Other antibodies included those recognizing kinesin family member 5B (KIF5B) (sc-133184), mitochondrial Rho GTPase 1 (Rho-T1) (sc-398520), Dynactin1 (sc-365054), and dynein light chain LC8-type 1 (DYNLL1) (sc-136287) (Santa Cruz Biotechnology, Santa Cruz, CA, U SA).Horseradish peroxidase (HRP)-conjugated goat anti-rabbit immunoglobulin G (Abcam) (ab6734). Other kits and reagents included, cadmium chloride (CdCl_2_) (Sigma-Aldrich, St. Louis, MO, USA); a Triglyceride (TG) assay kit and Total cholesterol (TC) assay kit (Nanjing Jiancheng Bioengineering Institute, Nanjing, China); Nocodazole (Noc) and Cytochalasin D (Cytd) (MedChemExpress, Monmouth Junction, NJ, USA); *GSK3B* (GSK-3β), *DCTN1* (Dynactin1), and *SQSTM1* (P62)-specific small interfering RNAs (siRNAs; RiboBio Corporation, Guangzhou, China); and a Cell Counting Kit-8 (CCK8; Yeasen BioTechnology, Shanghai, China).

### Animals and treatment

Albumin (Alb)-cre mice (used to generate conditional deletions in hepatocytes) were obtained from Cyagen Biosciences (Santa Clara, CA, USA) All mice were maintained on a C57BL/6J background. The mice were male, and were 8 weeks old. The adeno-associated virus vector AAV8-Rho-T1 was injected through the tail vein, and the mice were subsequently housed in a suitable environment (24 °C) for one month to detect RHOT1 expression levels. The 32 mice were evenly divided into four groups: (1) AAV8-Negative Control (AAV8-NC) group [double distilled water (DDW) intraperitoneally (i.p.)], (2) AAV8-NC + Cd group (1 mg/kg CdCl_2_, i.p.), (3) AAV8-Rho-T1 (DDW, i.p.), and (4) AAV8-Rho-T1 + Cd group (1 mg/kg CdCl_2_, i.p.). All mice had ad libitum access to food water and were maintained in a specific-pathogen-free facility with a 12 h:12 h light: dark cycle. Animals were randomly assigned at eight mice/group per experiment. After 2 weeks of exposure, the mice were humanely killed for tissue harvest using carbon dioxide asphyxiation. This study was approved by the Institutional Animal Care and Use Committee of Yangzhou University and carried out according to the Guide for the Care and Use of Laboratory Animals of the National Research Council (approval ID: SYXK (Su) 2022- 0044).

### Cell culture and processing

The alpha mouse liver 12 (AML12) cell line (AML12, ATCC^®^CRL-2254™, USA)was cultured in Dulbecco’s modified Eagle’s medium (DMEM)/F-12 supplemented with 1% Insulin-Transferrin-Selenium-Sodium Pyruvate (ITS-A), 10% fetal bovine serum (FBS), 100 U/ml penicillin, and 100 mg/ml streptomycin, and maintained at 37 °C with 5% CO_2_.

### Mitochondrial tracing in living cells

pCMV-Mito-AT1.03 is a tool plasmid containing a cytomegalovirus (CMV) promoter for the expression of the AT1.03 protein (AT1.03 protein is an ATP indicator based on the ε subunit of FoF1-ATP synthetase) with a mitochondrial localization signal (MLS) in mammalian mitochondria as a mitochondrial fluorescent probe. AML12 cells were seeded in a 35 cm confocal dish and when the cell density reached 60%, pCMV-Mito-AT1.03 was transferred into the cells using Lipofectamine 3000 (Invitrogen, Cambridge, MA, USA). After 12 h, the fusion protein was observed under a fluorescence microscope. Cells expressing fusion proteins were then transferred to a live-cell workstation to observe mitochondrial dynamics (AXIO observer 7, Carl Zeiss, Germany).

### Mito-tracker and CFDA-SE staining

Mito-Tracker and CFDA-SE were diluted with medium to form a staining working solution and stored in the dark for later use. AML12 cells were seeded in a petri dish and grown to 50% confluence. The cells were then immersed in the staining working solution at 37 °C for 20 min. The staining solution was aspirated off, the cells were washed three times with phosphate-buffered saline (PBS), fresh medium was added, and the cells were imaged under a fluorescence microscope.

### Scanning electron microscopy and transmission electron microscopy

AML12 cells were seeded in 24-well cell glass coverslip, and when the cells grew to 70%, the medium containing different concentrations of Cd solution was replaced with the basal medium, and culture was continued for 12 h. The medium was discarded, the cells were washed with PBS twice, 2.5% glutaraldehyde was added to fix the cells at room temperature for 2 h, the glutaraldehyde was discarded, and the cells were washed with PBS thrice. The cells were then dehydrated through an ethanol gradient, with 15 min intervals between different concentrations. The sample was freeze-dried, sprayed with gold, and then observed under a scanning electron microscope (GeminiSEM 300, Carl Zeiss, Germany). For transmission electron microscopy, mouse liver tissue was cut into 1 × 1 mm blocks, the blocks were cleaned using PBS, fixed using 2.5% glutaraldehyde for 2 h at room temperature, dehydrated, embedded, and sectioned on an ultramicrotome. The sections were double-stained using 3% uranium acetate and lead citrate, and then observed on a transmission electron microscope (HT7800, HITACHI, Japan).

### Establishment of a co-culture system for cells

In this study, we used Transwell assays to establish two cell culture models. 1. A group of Mito-tracker-labeled cells was seeded inside the Transwell membrane for testing. At the same time, a colony of unlabeled cells was inoculated in the internal chamber of the dish. When the cells were 80% confluent, the basal medium was replaced with a medium containing Cd. After continuing the culture for 12 h, the Transwell was removed, washed with PBS twice, and the cell in the dish were measured using fluorescence microscopy. 2. A group of Mito-tracker-labeled cells was seeded on the outside of the Transwell membrane, another group of cells labeled with CFDA-SE was seeded in inside the Transwell membrane. The two groups of cells were cultured together, and after the cells grew to 80%, the basal medium was replaced with medium containing Cd. After continuing culture for 6 h, the Transwell chamber was washed twice with PBS, the cells on the outside of the Transwell membrane were removed using a cell scraper, the Transwell chamber was washed twice with PBS, and the fluorescence changes in the cells inside the Transwell membrane were observed using fluorescence microscopy.

### Flow cytometry

In this study, two groups of cells pre-stained CFDA-SE and Mito-tracker were mixed in cell culture flasks, centrifuged, collected, washed with PBS, centrifuged, collected, and observed by flow cytometry (LSRFortessa, BD Biosciences, USA). Subsequently, we established two cell models: 1. A group of CFDA-SE pre-stained cells was seeded in a 60 mm cell culture dish and grown to 70% confluence. Cd solution was added to the medium and cultured was continued for 12 h. The medium was removed and the cells were washed with PBS twice. Another group of Mito-tracker pre-stained cells was also added to a dish in a similar proportion, fresh medium was added, and culture was continued for 12 h. The cells were incubated with trypsin without EDTA for 40 s, collected by centrifugation, filtered, centrifuged again, and subjected to flow cytometry. 2. In the other model the cell labeling was the opposite, i.e., CFDA-SE-stained cells are added to the Mito-tracker pre-stained cell population to measure the co-staining rate of the cells.

### Oil red O staining

Cell samples were fixed using 4% paraformaldehyde for 15 min, washed with PBS twice, added with staining wash solution to cover the cells for 20 s, after which the staining wash was discarded. Oil red O staining solution was added to the cells, incubated for 30 s, discarded, and staining wash solution was added and incubated for 30 min. The staining washing solution was then aspirated off, the cells were washed three times using PBS for each 3 min each time, and the cells were observed under the microscope. Tissue samples were fixed, dehydrated, embedded in optimal cutting temperature (OTC) resin, and sectioned to make frozen section. The staining wash solution was used to cover the sections for 20 s, the sections were immersed in the staining working solution, and stained for 20 min. The staining solution was aspirated off, the sections were covered with staining wash solution, incubated for 20 s, washed twice with PBS, and observed under the microscope.

### BODIPY staining

BODIPY was made into a staining working solution. For cell samples, 4% paraformaldehyde fixation was carried out for 15 min, the cells were washed twice with PBS for 2 min each time, and then incubated with staining working solution for 20 min. The staining working solution was aspirated off, the cells were washed with PBS, and finally, Hoechst staining solution was added and incubated for 10 min. The cells were then washed with PBS three times before being imaged under the fluorescence microscope.

### Hematoxylin–eosin staining

Part of the mouse liver was cut off at the same position in each mouse, trimmed, added to the fixative solution (4% paraformaldehyde), and fixed for 24 h. The fixative was discarded, and the tissue was washed, dehydrated through an ethanol concentration gradient, made transparent using a xylene solution, and impregnated with wax. The tissue was sectioned using a microtome and attached to a slide. The sections were then stained with hematoxylin for 10 min, rinsed twice under running water, stained using eosin staining solution for 1 min, rinsed twice under running water, and allowed to air dry. The dried sections were dehydrated, made transparent, and imaged under the microscope.

### Western blotting analysis

Cells grown on dishes were collected using a cell scraper, centrifuged, lysed in Radioimmunoprecipitation assay (RIPA) Lysis Buffer at 4 °C for 30 min, and treated with ultrasound twice for 5 s each time. The samples were centrifuged at 12,000 × *g* for 10 min, the supernatant was retained, and the protein concentration in the supernatant was determined using a bicinchoninic acid (BCA) kit (Yeasen BioTechnology, Shanghai, China). The different samples were adjusted to the same protein concentration with loading buffer, and placed at 100 °C for 8 min. The proteins were then separated using sodium dodecyl sulfate–polyacrylamide gel electrophoresis and transferred to polyvinylidene difluoride membranes (Millipore, Billerica, MA, USA). The membranes were subjected to blocking, washing with Tris Buffered Saline with Tween^®^ 20 (TBST), primary antibody incubation, and secondary antibody incubation, after which the densities of the immunoreactive protein bands were measured using Image Laboratory software (Bio-Rad, Hercules, CA, USA).

### Immunofluorescence

AML12 cells were seeded on 24-well cell glass coverslips, and then subjected to a series of treatments. The medium was then discarded, the cells were washed with PBS twice, fixed for 15 min in 4% paraformaldehyde, washed with PBS twice, and blocked for 2 h in blocking fluid (3% BSA solution containing 1% Triton X-100). The blocking fluid was removed and the cells were incubated with primary antibodies at 4 °C for 12 h, and then with the appropriate secondary antibodies at room temperature for 2 h, and washed with PBS three times. The cells could also be further co-stained with other staining agents (e.g., Tubulin-tracker, Phalloidin, Hoechst). After staining, anti-quencher was added, the coverslips were sealed, and then observed under a confocal microscope (TCS SP8 STED, Leica, Germany). Immunofluorescent staining of tissue samples was based on frozen sections. 0.5% TritonX-100 drops were added to the tissue sections, incubated at room temperature for 15 min, and then the TritonX-100 was discarded. The sections were blocked in PBS containing 10% normal goat serum at room temperature for 1 h. Primary antibodies were then added, incubated overnight at 4 °C, and then washed with PBS three times for 5 min each time. Then, different fluorescently labeled secondary antibodies were added, incubated at room temperature for 2 h, washed for 5 min, incubated with Hoechst in the dark for 10 min, washed with PBS three times, covered, and imaged under a fluorescence microscope.

### Isolation of mitochondria

Mitochondrial isolation kits were used to isolate mitochondria within cells based on the manufacturer's requirements. Exogenous mitochondrial origin with untreated AML12 cells.Briefly, treated cells digested using pancreasin, centrifuged, and collected. The mitochondrial separation reagent was added, and then the solution was transferred to a glass homogenizer, and homogenized 30 times. The liquid in the homogenizer was transferred to a 1.5 ml centrifuge tube, centrifuged at 600 × *g* at 4 °C for 10 min, and the supernatant was collected. The supernatant was centrifuged at 11,000 × *g* at 4 °C for 10 min, and the precipitate comprised the extracted mitochondria, which were resuspended in mitochondrial storage solution, and preserved for later use.

### TG and TC content determination

Tissue samples were weighed and a certain weight of tissue was added with normal saline at 1:9, homogenized, centrifuged at 2500 r/min for 10 min, and the supernatant was retained for measurement. Cell samples were made into a cell suspension using normal saline, disrupted using ultrasound, centrifuged, and the supernatant was retained for measurement. The TG and TC contents in the supernatants were determined according to the protocol of the corresponding test kit.

### Micro mitochondrial respiratory chain complex I, III, IV, and V Ca^2+^-Mg^2+^-ATPase/ATP content determination

The mitochondrial respiratory chain samples were prepared as follows: 0.1 g of tissue was weighed or 5 million cells were collected, and extracted to make a tissue suspension or cell suspension. The suspension was transferred to a glass homogenizer, collect the liquid after homogenizing, and centrifuged. The supernatant was transferred into a 1.5 ml centrifuge tube, centrifuged, the pellet was resuspended in the extraction buffer, and the cells/tissue were broken ultrasonically. The resulting liquid is used for subsequent activity determination. The ATP content and Ca^2+^-Mg^2+^-ATPase activity could be measured without homogenization. The assays were performed according to the manufacturer’s protocol.

### Co-immunoprecipitation

First, cells were digested and collected, added with a Cell lysis buffer for CO-IP (Beyotime Biotechnology, Shanghai, China) on ice for 30 min, and centrifuged at 4 °C and 12,000 × *g* for 15 min. Part of the supernatant was collected to prepare control protein samples. Then, 2 μg of the corresponding antibody was added to the remaining supernatant, and incubated overnight at 4 °C. At the same time, the magnetic beads were resuspended and cleaned with PBS (noting the damage to the beads during washing), centrifuged, placed on the magnetic stand, and the liquid was removed. The collected supernatant was added to resuspend the magnetic beads, which were incubated at room temperature for 2 h, Washed three times with the lysate, added with loading buffer, and denatured at high temperature for 10 min before electrophoresis..

### Cell viability assay

The CCK8 assay kit was used to detect cell viability. First, the required CCK8 working solution (CCK8 solution and medium 1:9 configuration) was calculated according to the number of samples, and protected from light. Cells were seeded in 96-well plates, and after a series of treatments, the medium was discarded and the CCK8 working solution was added. After incubation for 1 h, the OD value of each well was determined at 450 nm.

### Small interfering RNA transfection

The siRNA specific for GSK-3β, Rho-T1, Dynactin1, and P62 was synthesized by RiboBio Corporation (Guangzhou, China). AML12 cells were seeded in 6-well or 24-well plates. At 40% confluence, the cells were cultured in fresh medium without penicillin and streptomycin, together with transfection reagent (Polyplustransfection, Illkirch, France) and 20 nM GSK-3β, Rho-T1, Dynactin1,and P62 siRNA or negative control siRNA for 24 h, according to the manufacturer’s protocols. Then, the cells were subjected to other treatments as required by the experiments.

### Statistical analysis

The data from at least three independent experiments were analyzed statistically and expressed as the mean ± standard deviation (SD). GraphPad Prism 6 software (GraphPad Software Inc., La Jolla, CA, USA) analyzed the data using one-way analysis of variance ((ANOVA) (Scheffe’s SF test). *P* values less than 0.05 indicated a significant difference.

## Results

### Cadmium inhibits mitochondrial transfer between AML12 cells

**Fig. 1:**
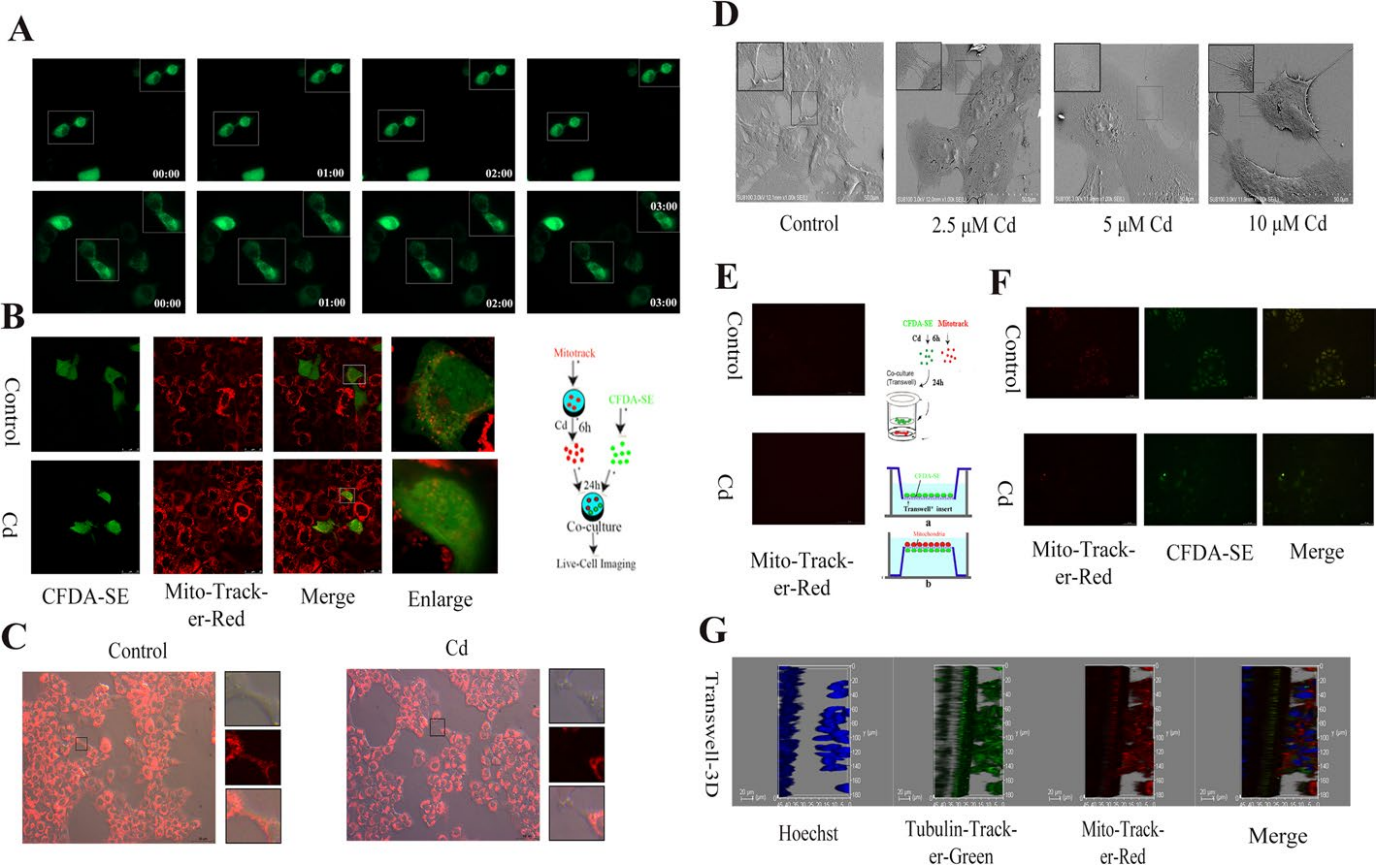
Cadmium inhibits mitochondrial transfer between AML12 cells. AML12 cells were treated with 5 μM Cd for 6 h. Live cell imaging of mitochondria (**A**), one group of CFDA-SE prestained cells was co-cultured with another population of Mito-tracker-Red prestained cells for confocal microscopy (**B**) Scale bar = 25 μm. Mito-tracker-Red with cell brightfield dual channel observation of mitochondrial transfer (**C**). Scale bar = 50 μm. AML12 cells were treated with 2.5, 5, and 10 μM Cd for 6 h, Scanning electron microscopy was used to observe intercellular tunneling nanotube structures (**D**). Scale bar = 50 μm. The Transwell co-culture system was used to detect the method of mitochondrial transfer (**E**, **F**). Scale bar = 50 μm. Confocal 3D imaging to detect intercellular mitochondrial transfer (**G**) Scale bar = 20 μm

To explore whether mitochondrial transfer also exists between hepatocytes, we established a variety of cell models. First, we constructed AML12 cells expressing a mitochondrial localization protein, as shown in the results of Fig. 1A, and live-cell imaging showed that mitochondria achieved cross-cell transfer (Additional file 2: Movie S1, Additional file 3: Movie S2). In a multicellular co-culture system, cells were pretreated with CFDA-SE and Mito-tracker-Red, and we observed labeled mitochondria (Fig. 1B) in the CFDA-SE pretreated cells. Mitochondrial labeling and scanning electron microscopy (SEM) were used to study the way mitochondria are transferred between cells. Superimposing Mito-Tracker-Red and cellular brightfield optical pathways, we found that a channel appeared between different cells and mitochondria were present within the channel (Fig. 1C). After Cd treatment, the cell-to-cell channels were thinned and no mitochondria were found. The SEM results showed more clearly that multiple channels with different morphologies were formed between the cells (Fig. 1D). Changes in the width of the intercellular channels occurred under treatment with different concentrations of Cd. Thicker channels were gradually replaced by thinner channels, and intercellular access was broken. To rule out the influence between cells in the same plane, we used Transwell chambers to establish a co-culture model using two groups of cells pretreated with CFDA-SE and Mito-tracker-Red, which were seeded on the plate and inside the Transwell membrane, respectively. Mito-tracker-Red fluorescence was not observed in the CFDA-SE pretreated cell populations (Fig. 1E) When CFDA-SE and Mito-tracker-Red pretreated cells were seeded on both sides of the Transwell membrane, Mito-tracker-Red fluorescence was observed in the CFDA-SE pretreated cell population, while after Cd treatment, Mito-tracker-Red fluorescence decreased in the CFDA-SE pretreated cell population (Fig. 1F). To rule out the superposition effect of fluorescence, we removed the treated Transwell membrane and placed it under a confocal microscope with a glass slide for multi-level scanning to form a 3D view (Fig. 1G). The results showed that CFDA-SE and Mito-tracker staining ran through the Transwell membrane.

### Mitochondrial transfer is bidirectional and associated with microtubules

**Fig. 2:**
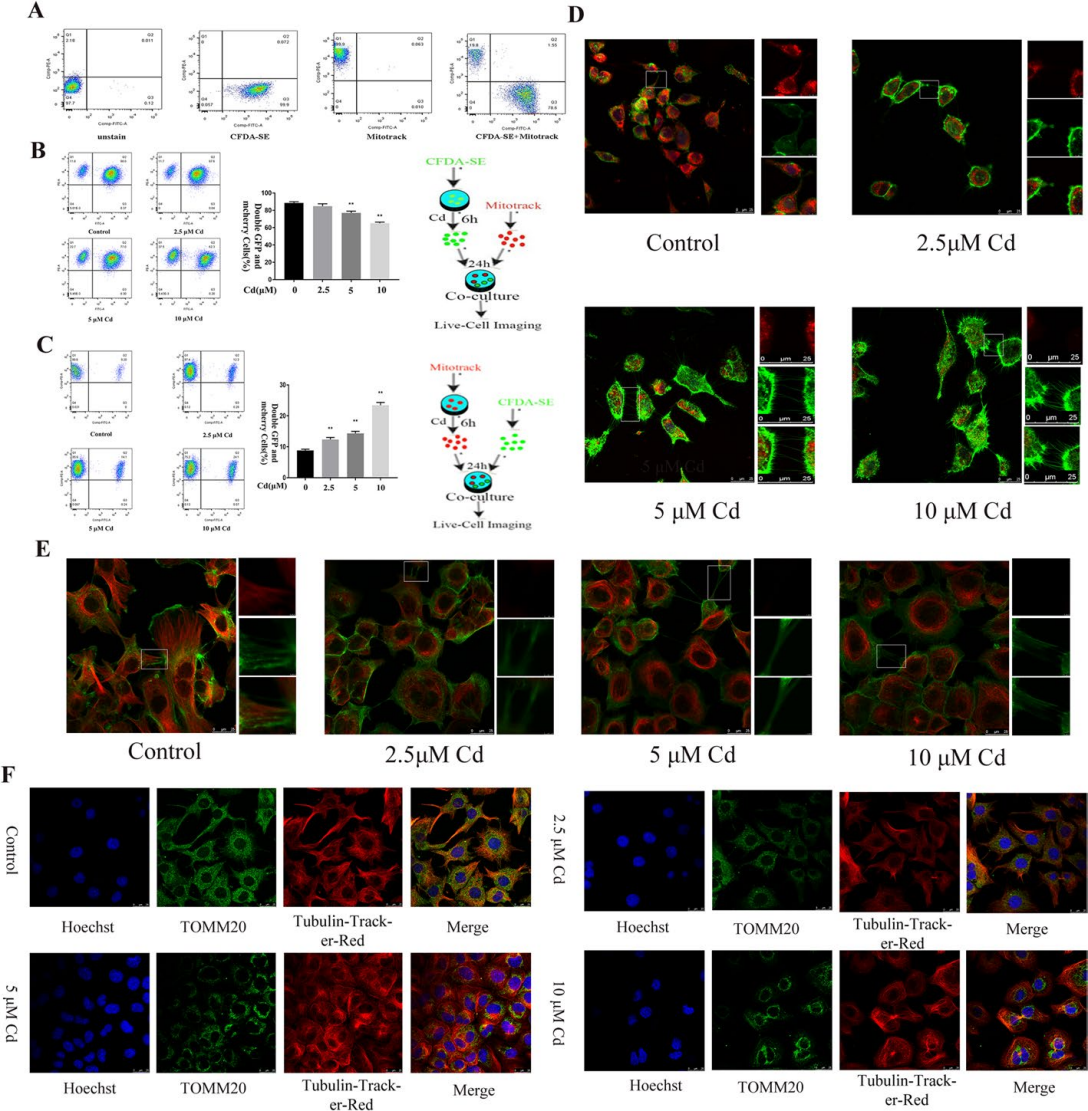
Mitochondrial transfer is bidirectional and associated with microtubules. AML12 cells were treated with 5 μM Cd for 12 h. One group of CFDA-SE prestained cells was co-cultured with another population of Mito-tracker-Red prestained cells for Flow cytometry to detect intercellular mitochondrial transfer efficiency (**A**–**C**). AML12 cells were treated with 2.5, 5, and 10 μM Cd for 6 h. Confocal microscopy was used to observe colocalization of phalloidin and Mito-tracker-Red (**D**). Scale bar = 25 μm. Tubulin-tracker and phalloidin co-staining to observe the effect of Cd on the cytoskeleton of AML12 cells (**E**). Scale bar = 25 μm. Confocal microscopy was used to observe colocalization of Tubulin-tracker-Red and Tomm20 (**F**). Scale bar = 25 μm

Flow cytometry was used to quantify the mitochondrial transfer efficiency. First, to rule out the risk of Mito-tracker-Red spread, we mixed two groups of cells that were pretreated with CFDA-SE and Mito-tracker-Red. The results demonstrated that the mixed cells showed a well-defined cell segmentation (Fig. 2A). Subsequently, to verify whether mitochondrial transfer is directional, we established two different co-culture models, one was to label untreated cells with mitochondria, and the result is shown in Fig. 2B, in which Cd reduced the mitochondrial transfer efficiency. The other was to label Cd-treated cells with mitochondria (Fig. 2C); however, interestingly, Cd increased the mitochondrial transfer efficiency. To verify whether mitochondrial transfer efficiency is time-dependent, we also tested mitochondrial transfer efficiency at 3 h and 6 h, and the results were consistent with those at 12 h (Additional file 1: Figure S1A-B). The cytoskeleton is the main component of tunneled nanotubes, which is involved in the transport of substances. Figure 2E shows that both microtubules and F-actin were present in AML12 intercellular tunneling nanotubes, but with the increase in the Cd concentration, the content of microtubules in the duct decreased and tended to show a perinuclear location. We then further co-localized mitochondria with actin filaments and microtubules, respectively. The results showed that Cd reduced the number of mitochondria in the tunneled nanotubes, and the mitochondria tended to be perinucleated, which was consistent with the effect of Cd on microtubules. However, Cd exposure showed a tendency to promote the growth of F-actin, which was inconsistent with mitochondrial dynamics (Fig. 2D, F).

### Cadmium disrupts mitochondrial respiratory chain function and fatty acid oxidation, and promotes hepatic lipid accumulation

**Fig. 3:**
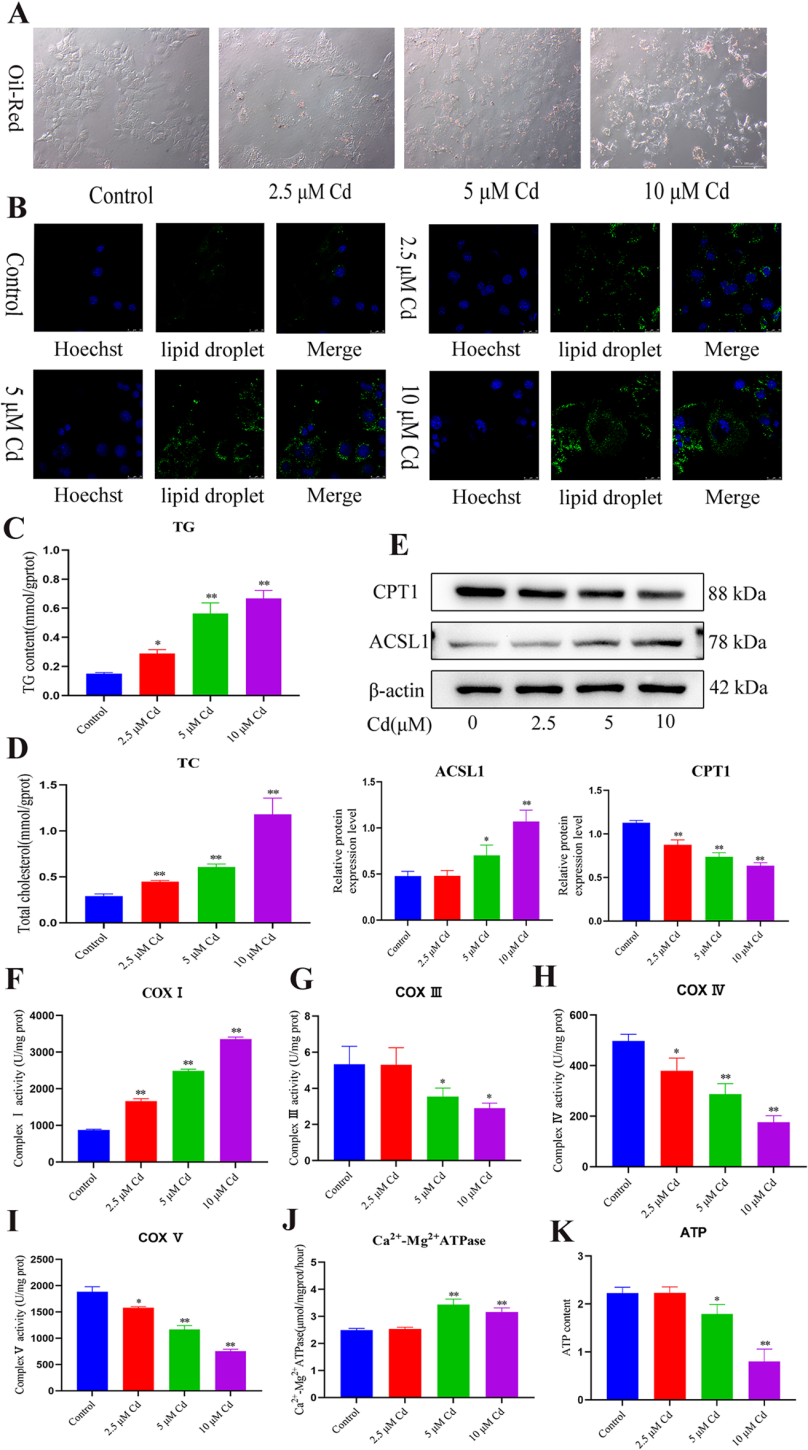
Cadmium disrupts mitochondrial respiratory chain function and fatty acid oxidation, and promotes hepatic lipid accumulation. AML12 cells were treated with 2.5, 5, and 10 μM Cd for 6 h. Oil red O (**A**) and BODIPY (**B**) were used to observe intracellular lipid droplets. Scale bar = 100 μm. Scale bar = 25 μm. TG and TC contents were detected (**C, D**). Levels of CPT1and ACSL1 were determined using western blotting (**E**). Results are shown as the mean ± SD (*n* = 3). Compared with the control group, *P < 0.05, **P < 0.01. The enzyme activities of the mitochondrial respiratory chain I (**F**), III (**G**), IV (**H**), and V (**I**) were examined. ATP content and Ca^2+^-Mg^2+^-ATPase were examined (**J**, **K**). Results are shown as the mean ± SD (*n* = 3). Compared with the control group, *P < 0.05, **P < 0.01

Oil red O and BODIPY probes were used to study the effect of Cd on lipid accumulation in the liver. The results showed that with the increase in Cd concentration, the staining intensity of oil red O and BODIPY fluorescence increased in a concentration-dependent manner (Fig. 3A, B). TG and TC levels were also increased in a Cd concentration-dependent manner (Fig. 3C, D). The western blotting results showed that Cd significantly reduced the level of CPT1 and increased the level of ACSL1 (Fig. 3E). Mitochondria are closely related to fat metabolism; therefore, we examined the effect of Cd on mitochondrial respiratory chain function. The results showed that Cd reduced the activity of mitochondrial respiratory chain complex III, IV, and V and ATP content, and increased the activity of mitochondrial respiratory chain I and Ca^2+^-Mg^2+^-ATPase (Fig. 3F–I).

### Disruption of microtubules inhibits mitochondrial transfer and promotes lipid accumulation

**Fig. 4:**
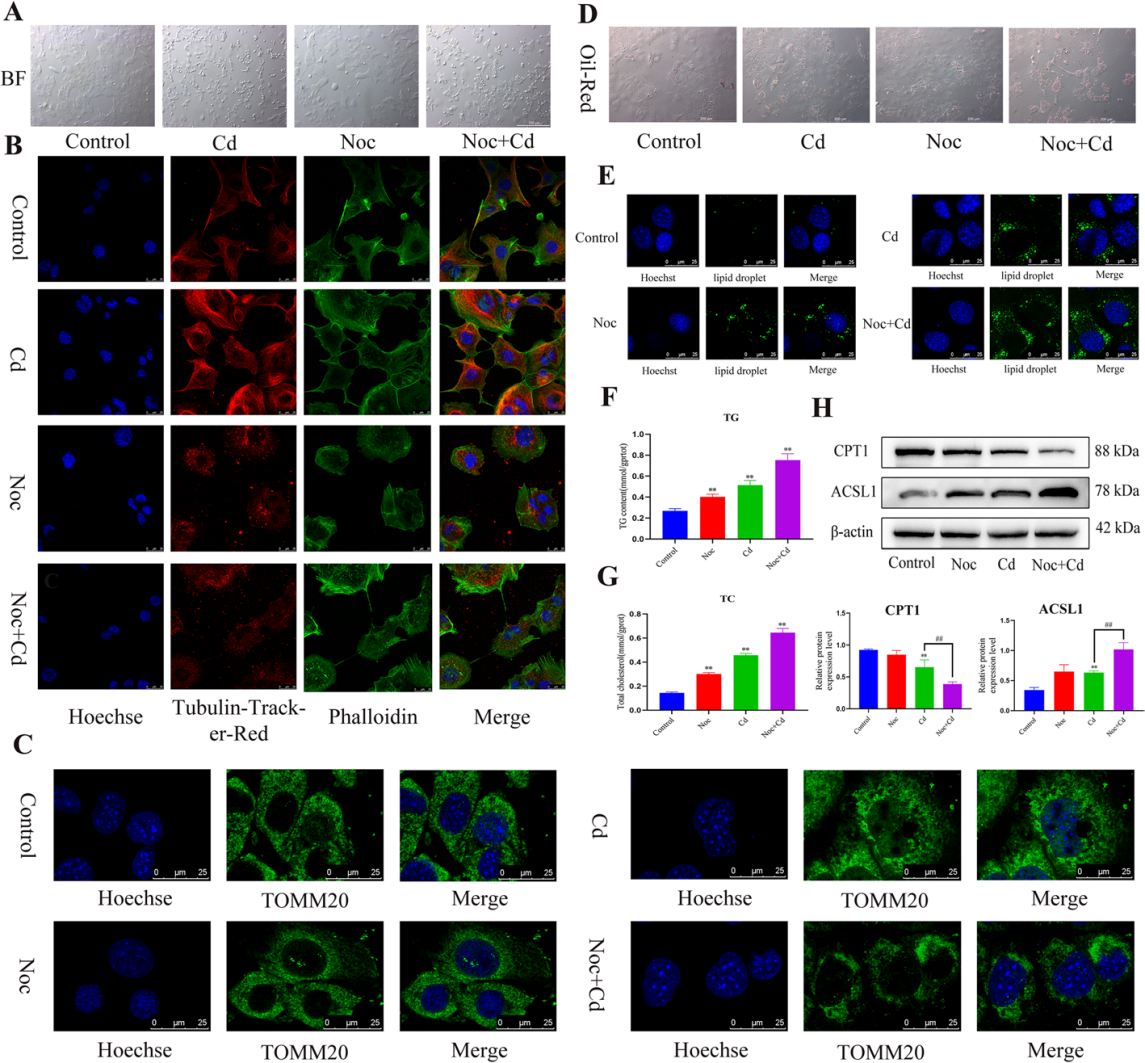
Disruption of microtubules inhibits mitochondrial transfer and promotes lipid accumulation. AML12 cells were exposed to 5 μM Cd and/or 2 μM Noc for 6 h. Brightfield (BF) microscopy was used to detect the effect of Cd on cell morphology (**A**). Scale bar = 100 μm. Tubulin-Tracker-Red and phalloidin co-staining were used to observe the cytoskeleton (**B**). Scale bar = 25 μm. Immunofluorescence was used to observe mitochondrial morphology (**C**). Scale bar = 25 μm. Oil red O (**D**) and BODIPY (**E**) were used to observe intracellular lipid droplets. TG and TC contents were detected (**F, G**). Levels of CPT1and ACSL1 were determined using western blotting (**H**). Results are shown as the mean ± SD (*n* = 3). Compared with the control group, *P < 0.05, **P < 0.01. Compared with the Cd group, ^#^*P* < 0.05, ^##^*P* < 0.01

To further verify whether the effect of Cd on mitochondrial transfer is related to changes in microtubules, Noc was co-treated with Cd. The results showed that after Noc treatment, the cell morphology tended to be rounded and long-distance contact between cells was blocked (Fig. 4A). Microtubule and F-actin staining showed that after Noc treatment, the filamentous structure of the microtubules was destroyed, forming numerous spot-like structures dispersed in the cell, and intercellular mitochondrial transfer was inhibited (Fig. 4C) (Additional file 1: Figure S3, S4), but did not have a significant effect on the structure of actin filaments (Fig. 4B). We then used Cytd, an inhibitor of F-actin, with Cd treatment, and Tubulin-tracker-Red and Phalloidin staining showed that after Cytd treatment, the filamentous structure of F-actin was broken and concentrated in the cytoplasm; however, interestingly, Cytd promoted the growth of microtubules and was mainly concentrated in the tunneled nanotubes (Additional file 1: Figure S2A). Co-staining of TOMM20 with Phalloidin also showed the same trend, with the destruction of the F-actin structure destruction and the promotion of mitochondrial transfer (Additional file 1: Figure S2B). SEM to observe the tunneling nanotubes showed a significant increase in the tunneling nanotubes structure after Cytd treatment (Additional file 1: Figure S2C). However, whether cytoskeleton-mediated mitochondrial transfer is involved in lipid metabolism is unclear; therefore, AML12 cells were incubated with Noc and Cytd, respectively, and the results showed that Noc co-treatment with Cd increased the intensity of oil red O staining and BODIPY fluorescence intensity compared with Cd alone (Fig. 4D, E). Cytd co-treatment with Cd reduced the intensity of oil red O staining compared with Cd alone (Additional file 1: Figure S2D). Compared with Cd group, the Noc + Cd group increased the content of TG and TC (Fig. 4F, G). Western blotting for CPT1 and ACSL1 showed that Noc exacerbates Cd-induced loss of CPT1 and accumulation of ACSL1 (Fig. 4H).

### The GSK-3β-Tau pathway mediates cadmium-induced disruption of microtubules

**Fig. 5:**
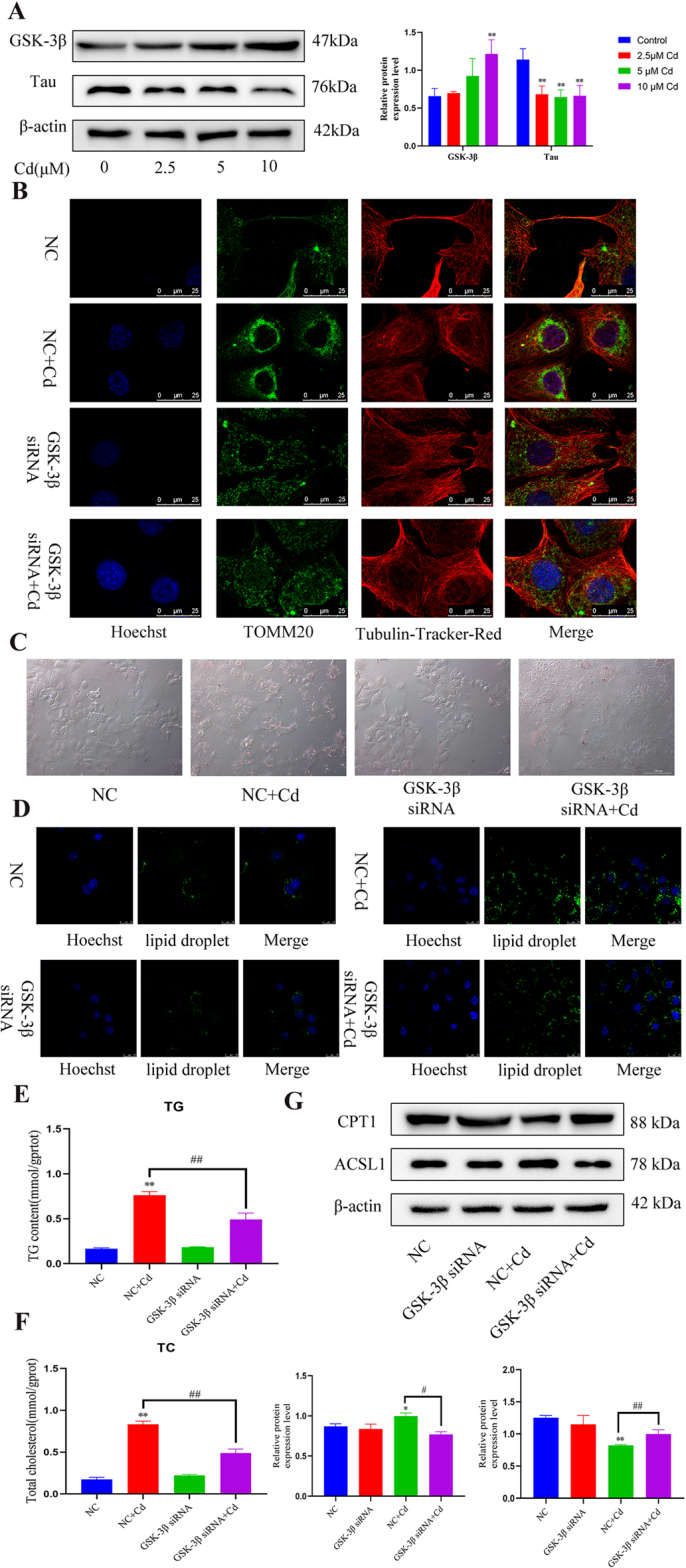
The GSK-3β-Tau pathway mediates cadmium-induced disruption of microtubules. AML12 cells were treated with 2.5, 5, and 10 μM Cd for 6 h. Levels of GSK-3β and Tau were determined using western blotting (**A**). Results are shown as the mean ± SD (*n* = 3). Compared with the control group, *P < 0.05, **P < 0.01. Based on treatment with *GSK3B* siRNA or NC siRNA for 24 h, AML12 cells were treated with or without Cd for 6 h. TOMM20 and Tubulin-Tracker-Red co-staining to detect mitochondrial transfer (**B**). Scale bar = 25 μm. Oil red O (**C**) and BODIPY (**D**) were used to observe intracellular lipid droplets. Scale bar = 25 μm. TG and TC contents were detected (**E, F**). Levels of CPT1and ACSL1 were determined using western blotting (**G**). Results are shown as the mean ± SD (*n* = 3). Compared with the control group, *P < 0.05, **P < 0.01. Compared with the Cd group, ^#^*P* < 0.05, ^##^*P* < 0.01

To explore the specific mechanism of Cd inhibition of microtubules, we first tested the expression level of GSK-3β and Tau. The western blotting results showed that Cd increased the level of GSK-3β and decreased the level of Tau in a concentration-dependent manner (Fig. 5A). Subsequently, cells were co-treated with *GSK3B* siRNA and Cd. TOMM20 staining appeared in the perinuclear region, and the microtubule structure was disordered and showed a shrinking trend, thus *GSK3B* siRNA co-treatment with Cd significantly alleviated the Cd-induced changes in mitochondrial position and the microtubule structure (Fig. 5B). We further examined some indexes related to lipid metabolism, and *GSK3B* siRNA co-treatment with Cd reduced the oil red O staining intensity (Fig. 5C) and the BODIPY fluorescence intensity (Fig. 5D). Compared with those in the Cd group, the TG and TC contents also showed a decreasing trend (Fig. 5E, F). Western blotting for CPT1 and ACSL1 showed that silencing *GSK3B* alleviated Cd-induced protein changes (Fig. 5G).

### Cadmium inhibits KIF5B-Rho-T1-driven mitochondrial transfer and increases lipid accumulation

**Fig. 6:**
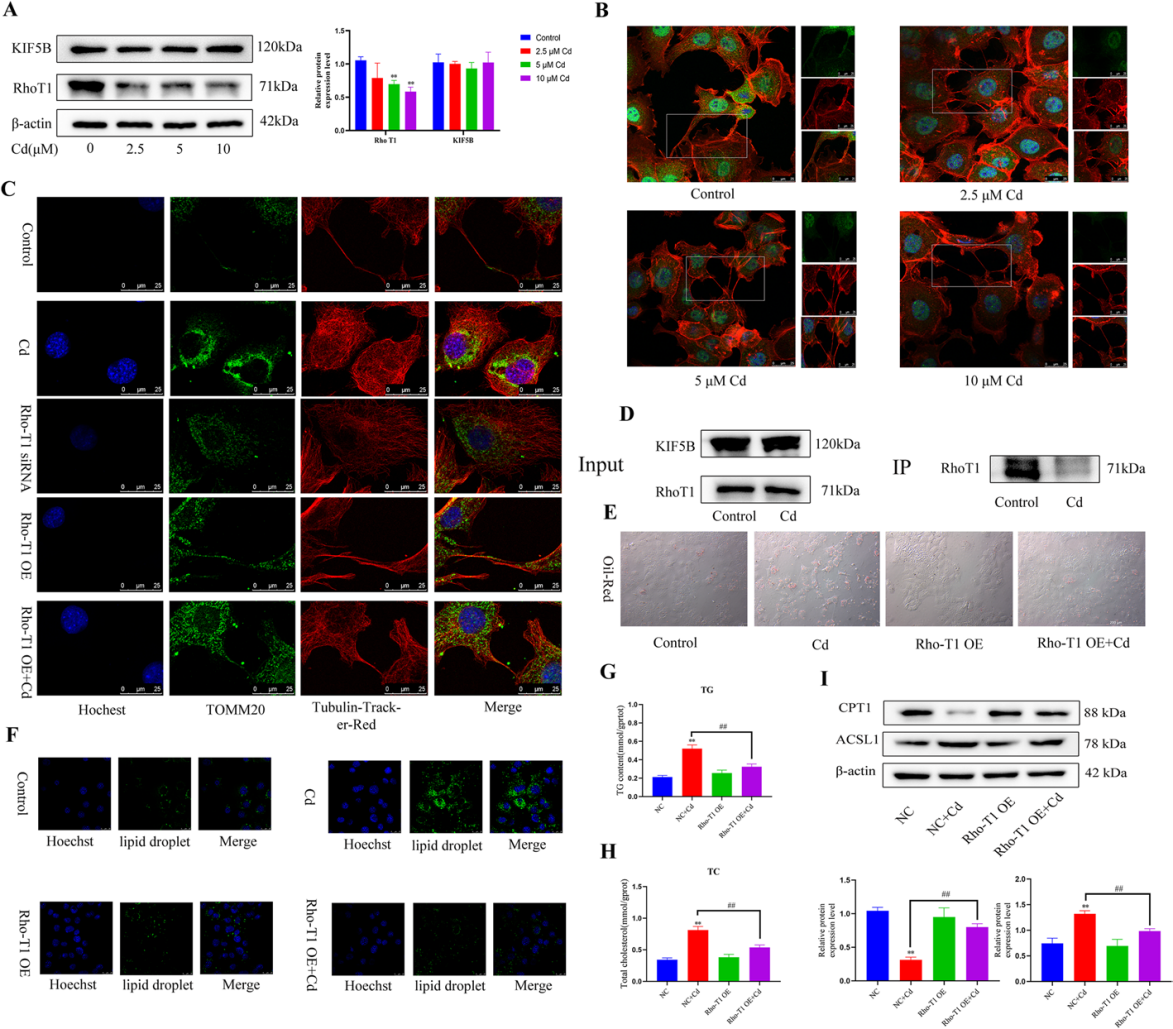
Cadmium inhibits KIF5B-RhoT1-driven mitochondrial transfer and increases lipid accumulation. AML12 cells were treated with 2.5, 5, and 10 μM Cd for 6 h. Levels of KIF5B and RhoT1 were determined using western blotting (**A**). Results are shown as the mean ± SD (*n* = 3). Compared with the control group, *P < 0.05, **P < 0.01. RhoT1 and phalloidin were co-stained to observe RhoT1 intracellular localization (**B**). Scale bar = 25 μm. Based on treatment with *KIF5B* siRNA or *KIF5B* OE plasmid for 24 h, AML12 cells were treated with or without Cd for 6 h. TOMM20 and Tubulin-Tracker-Red co-staining to detect mitochondrial transfer (**C**). Scale bar = 25 μm. AML12 cells were treated with 5 μM Cd for 6 h. Co-immunoprecipitation detection of KIF5B and RhoT1 interactions (**D**). Based on treatment with *KIF5B* OE plasmid for 24 h, AML12 cells were treated with or without Cd for 6 h. Oil red O (**E**) and BODIPY (**F**) were used to observe intracellular lipid droplets. Scale bar = 200 μm. Scale bar = 25 μm. TG and TC contents were detected (**G, H**). Levels of CPT1and ACSL1 were determined using western blotting (**I**). Results are shown as the mean ± SD (*n* = 3). Compared with the control group, *P < 0.05, **P < 0.01. Compared with the Cd group, ^#^*P* < 0.05, ^##^*P* < 0.01

Motor proteins connect mitochondria to microtubules and drive the movement of mitochondria. To explore the effect of Cd on motor proteins, we first examined the effects of Cd on KIF5B and Rho-T1, and the results showed that KIF5B did not change; whereas Rho-T1 levels were significantly reduced after Cd treatment (Fig. 6A). We also found that Cd significantly reduced the interaction between KIF5B and Rho-T1 (Fig. 6D). Co-staining of Rho-T1 with Phalloidin Peptide showed that after Cd treatment, the fluorescence intensity of Rho-T1 in the intercellular duct decreased significantly, and with increasing Cd concentration, the fluorescence tended to aggregate in the nucleus (Fig. 6B). Subsequently, we overexpressed (OE) *Rho-T1* in Cd-treated cells, and compared them with the Cd group. In the *RhoT1* OE + Cd group he perinuclear aggregation phenomenon of mitochondria increased and intercellular mitochondrial transfer was promoted (Fig. 6C). We also tested lipid-related indexes, and the *RhoT1* OE + Cd group showed reduced oil red O staining intensity (Fig. 6E) and BODIPY fluorescence intensity (Fig. 6F) compared that in the Cd group. The contents of TG and TC in the cells also showed a decreasing trend (Fig. 6G, H). Western detected CPT1 and ACSL1 levels, and *RhoT1* OE alleviated the Cd-induced protein changes (Fig. 6I).

### P62-dynaction1-driven negative mitochondrial transport mediates cadmium-mediated inhibition of mitochondrial transfer

**Fig. 7:**
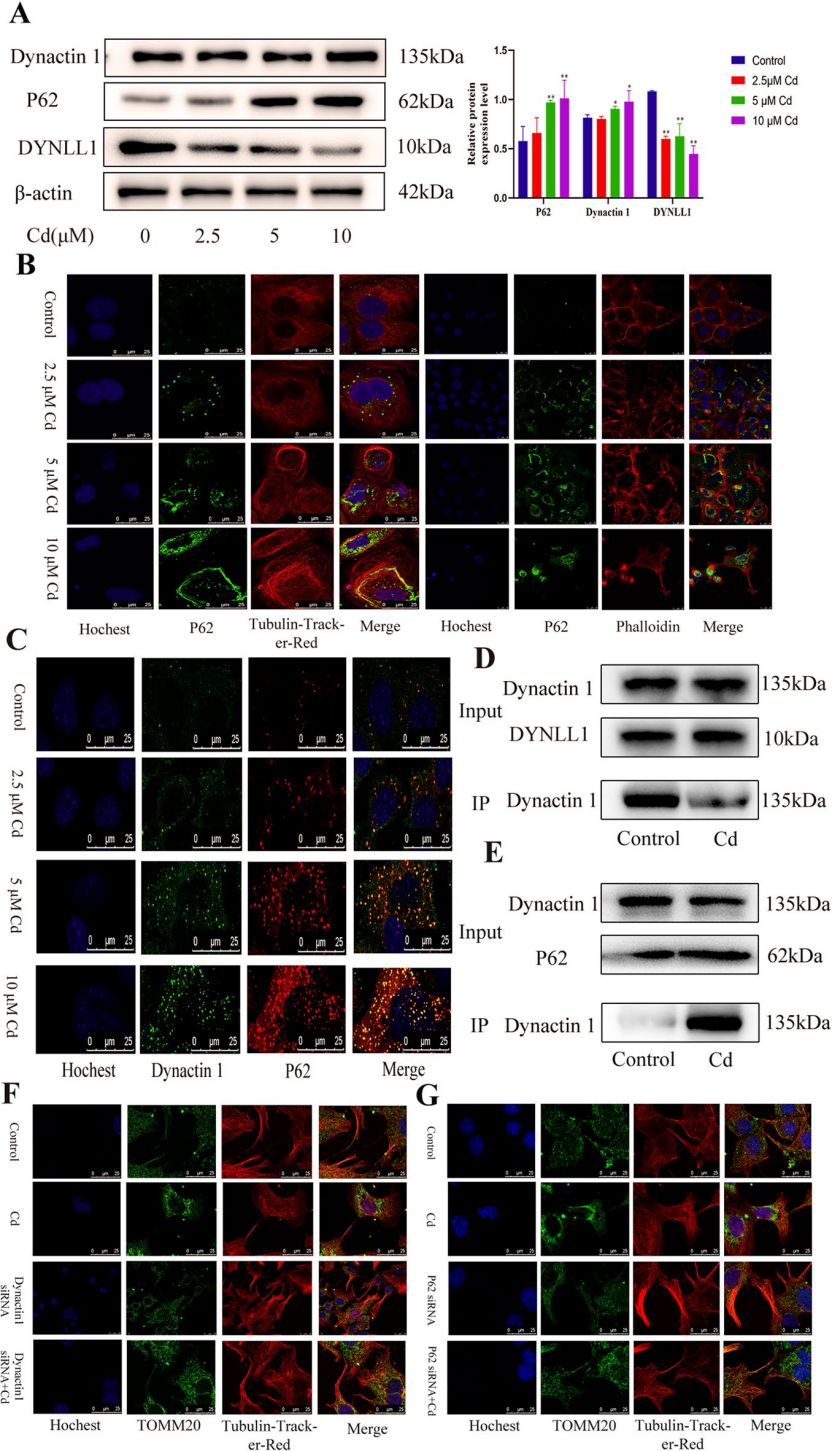
P62-Dynaction1-driven negative mitochondrial transport mediates cadmium-induced inhibition of mitochondrial transfer. AML12 cells were treated with 2.5, 5, and 10 μM Cd for 6 h. Levels of Dynactin 1, P62, and DYNLL1 were determined using western blotting (**A**). Results are shown as the mean ± SD (*n* = 3). Compared with the control group, *P < 0.05, **P < 0.01. P62, Tubulin-Tracker-Red, and phalloidin co-staining (**B**). Scale bar = 25 μm. Co-staining of P62 and Dynactin1 was observed (**C**). Scale bar = 25 μm. AML12 cells were treated with 5 μM Cd for 6 h. Co-immunoprecipitation detection of Dynactin1 and DYNLL1 interactions (**D**). Co-immunoprecipitation detection of Dynactin1 and P62 interactions (**E**). Based on treatment with Dynactin1 siRNA and P62 siRNA for 24 h, AML12 cells were treated with or without Cd for 6 h. TOMM20 and Tubulin-Tracker-Red co-staining to detect mitochondrial transfer (**F, G**). Scale bar = 25 μm

To further explore the specific mechanism of Cd-induced perinuclear mitochondrial aggregation, we examined the expression levels of P62, Dynactin1, and DYNLL1. Cd increased the levels of P62 and Dynactin1, but decreased the level of DYNLL1 (Fig. 7A). Subsequently, P62 was co-stained using Tubulin-tracker-Red and Phalloidin peptide, respectively, and the results showed that with the increase in Cd concentration, the fluorescence point of P62 increased significantly, which peaked under 10 μM Cd, forming a chain-like structure and aggregating around the nucleus. During this period, the P62 fluorescent coincided with the microtubules. There was no correlation between P62 fluorescence and the change in F-actin (Fig. 7B). We further detected the colocalization of P62 and Dynactin1. The immunofluorescence results showed that with the increase in Cd concentration, the fluorescence intensity of P62 and Dynactin1 increased, and increased fluorescent co-localization (Fig. 7C). To further explore the interactions among P62, Dynactin1, and DYNLL1, the interaction between Dynaction1 and DYNLL1 was first detected, and the co-immunoprecipitation results showed that Cd reduced the interaction between Dynaction1 and DYNLL1 (Fig. 7D). We then examined the interaction between P62 and Dynactin1, and found that Cd significantly increased the interaction between P62 and Dynactin1 (Fig. 7E). Finally, we explored the role of P62 and Dynactin1 in mitochondrial transfer, and T0MM20 and Tubulin-tracker-Red staining showed that siRNA inhibition of P62 and Dynactin1 expression alleviated Cd inhibition of mitochondrial transfer (Fig. 7F, G).

### Inhibition of P62 alleviates cadmium-induced lipid accumulation

**Fig. 8:**
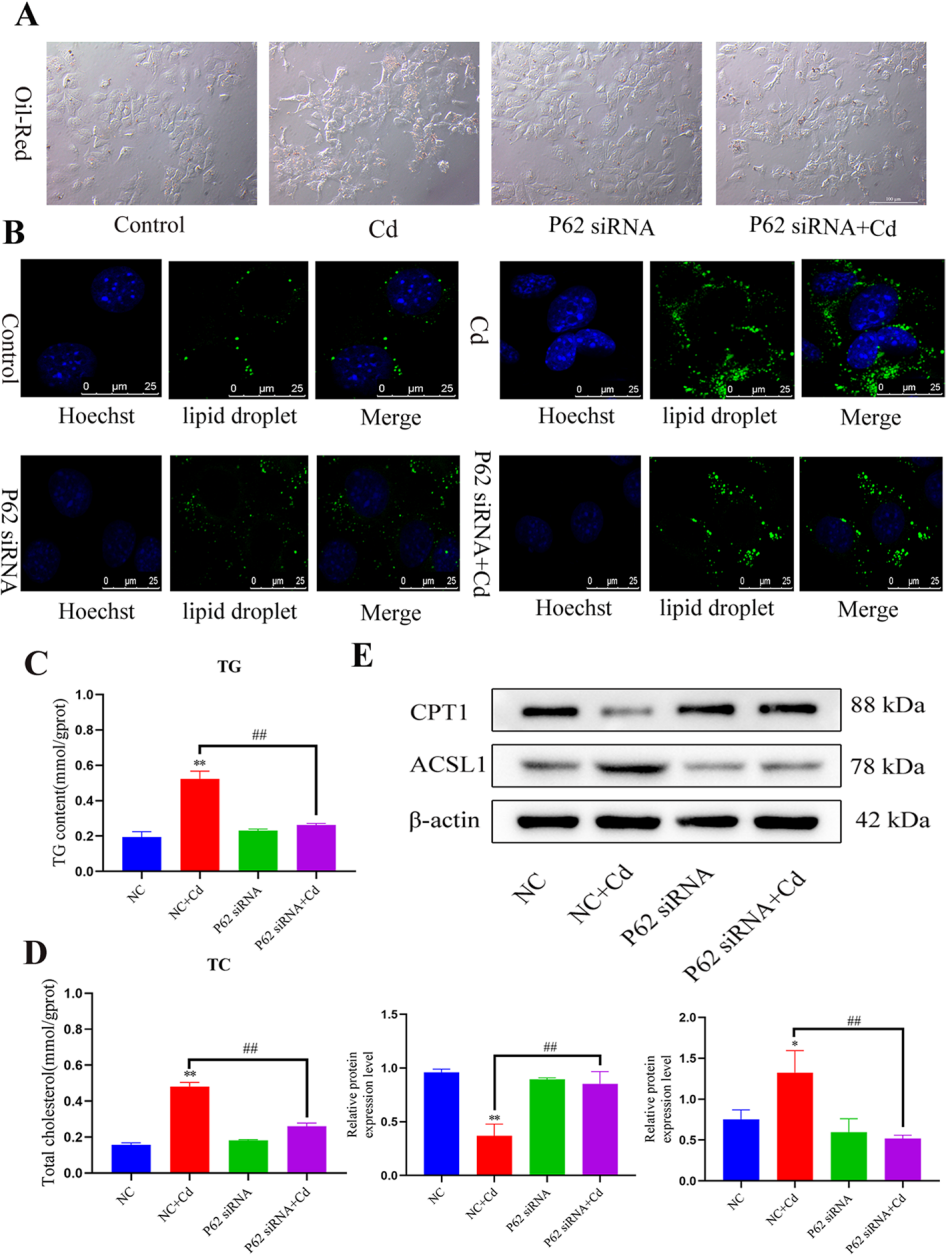
Inhibition of P62 alleviates cadmium-induced lipid accumulation. Based on treatment with *SQSTM1* (P62) siRNA or NC siRNA for 24 h, AML12 cells were treated with or without Cd for 6 h. Oil red O (**A**) and BODIPY (**B**) were used to observe intracellular lipid droplets. Scale bar = 200 μm. Scale bar = 25 μm. TG and TC contents were detected (**C**, **D**). Levels of CPT1and ACSL1 were determined using western blotting (**E**). Results are shown as the mean ± SD (*n* = 3). Compared with the control group, *P < 0.05, **P < 0.01. Compared with the Cd group, ^#^*P* < 0.05, ^##^*P* < 0.01

To study the effect of P62 on lipid accumulation, cells were co-treated with an *SQSTM1* (P62) siRNA and Cd. The results showed that Cd increased the oil red O staining intensity, and *SQSTM1* siRNA co-treatment with Cd decreased the oil red O staining intensity (Fig. 8A). The BODIPY fluorescence staining showed the same trend (Fig. 8B), and the *SQSTM1* siRNA + Cd co-treatment group had reduced the TG and TC contents compared those of the Cd group (Fig. 8C, D). Western blotting for CPT1 and ACSL1 levels showed that *SQSTM1*silencing alleviated the Cd-induced changes in protein levels (Fig. 8E).

### Exogenous mitochondria relieve cadmium-induced cell damage

**Fig. 9:**
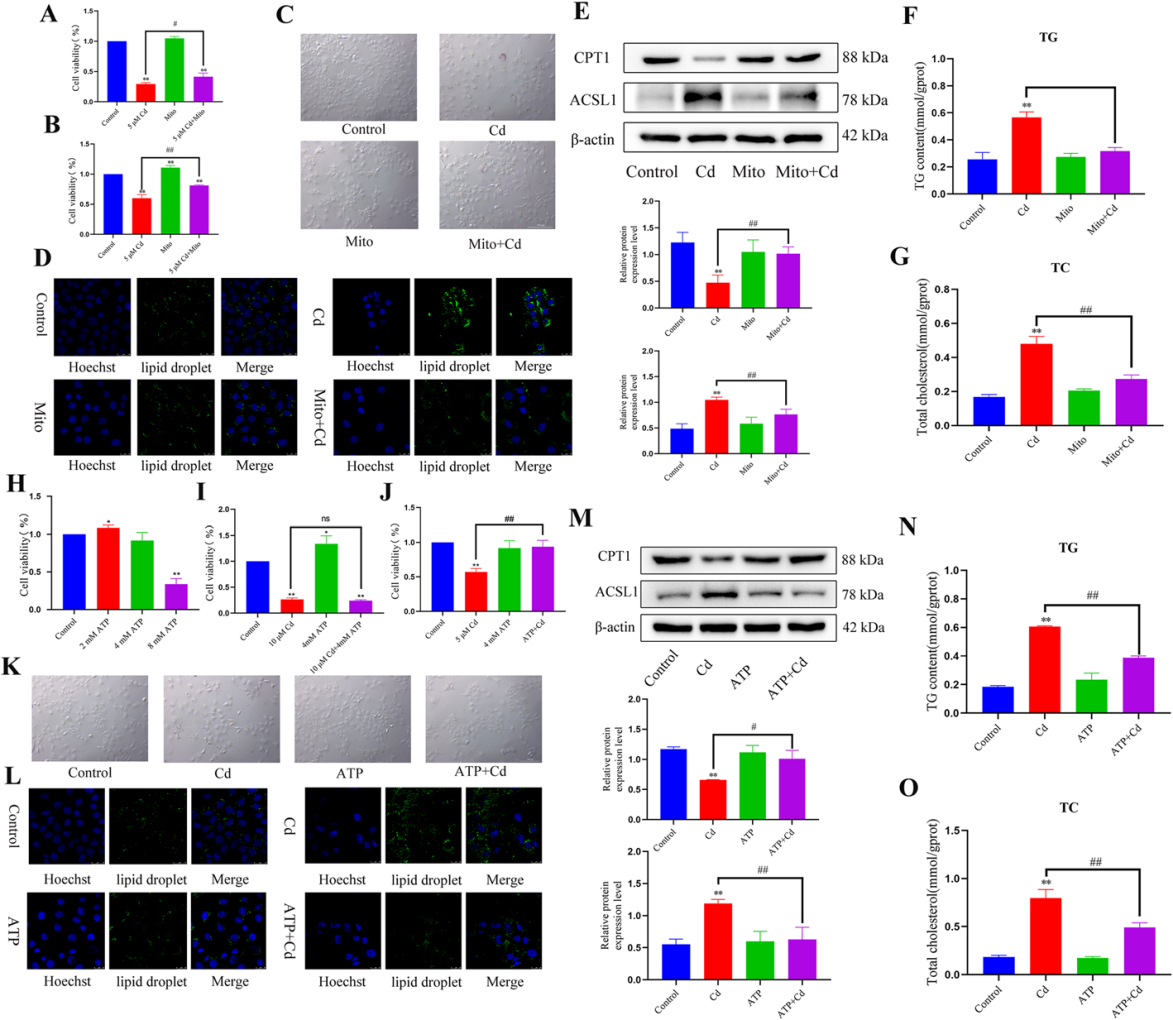
Exogenous mitochondria relieve cadmium-induced cell damage. AML12 cells were exposed to 5 μM Cd for 6 h. Subsequently, they were co-cultured with mitochondria for 12 h, and was used CCK8 to detect cell activity (**A**). After mitochondrial pretreatment for 6 h, AML12 cells were exposed to 5 μM Cd for 6 h. CCK8 detection of cell activity (**B**). Compared with the control group, *P < 0.05, **P < 0.01. Compared with the Cd group, ^#^*P* < 0.05, ^##^*P* < 0.01. After mitochondrial pretreatment for 6 h, AML12 cells were exposed to 5 μM Cd for 6 h. Oil red O (**C**) and BODIPY (**D**) were used to observe intracellular lipid droplets. Scale bar = 200 μm. Scale bar = 25 μm. Levels of CPT1and ACSL1 were determined using western blotting (**E**). TG and TC contents were detected (**F**, **G**). Results are shown as the mean ± SD (*n* = 3). Compared with the control group, *P < 0.05, **P < 0.01. Compared with the Cd group, ^#^*P* < 0.05, ^##^*P* < 0.01. AML12 cells were treated with 2, 4, and 8 mM ATP for 6 h. CCK8 was used to detect cell activity (**H**). ATP pretreatment 6 h, AML12 cells were exposed to 5 and 10 μm Cd for 6 h, and CCK8 detected cell activity (**I**, **J**). Compared with the control group, *P < 0.05, **P < 0.01. Compared with the Cd group, ^#^*P* < 0.05, ^##^*P* < 0.01. After ATP pretreatment for 6 h, AML12 cells were exposed to 5 μM Cd for 6 h. Oil red O (**K**) and BODIPY (**L**) were used to observe intracellular lipid droplets. Scale bar = 200 μm. Scale bar = 25 μm. Levels of CPT1and ACSL1 were determined using western blotting (**M**). TG and TC contents were detected (**N**, **O**). Results are shown as the mean ± SD (*n* = 3). Compared with the control group, *P < 0.05, **P < 0.01. Compared with the Cd group, ^#^*P* < 0.05, ^##^*P* < 0.01

To explore the fate of transferred mitochondria, we first added isolated mitochondria to Cd-treated cells. The results showed that mitochondria played a certain role in reversing the decrease of cell activity after Cd treatment (Fig. 9A). Another model was also established, in which cells were co-treated with mitochondria and Cd, and the results showed that mitochondria significantly alleviated the Cd-induced decrease in cell activity (Fig. 9B). We also measured the lipid content in the cells, which showed that exogenous mitochondria significantly reduced the intensity of oil red O and BODIPY fluorescence staining (Fig. 9C, D). Western blotting for CPT1 and ACSL1 levels showed that mitochondria alleviated the Cd-induced protein changes (Fig. 9E). Exogenous mitochondria also reversed the changes to the TG and TC contents induced by Cd (Fig. 9F, G). ATP is the most important product of mitochondria; therefore, we first determined the level of ATP that causes cell damage. The results showed that when the ATP concentration reached 8 mM, cell damage occurred (Fig. 9H). In this study, cells were co-treated with 4 mM ATP and Cd, and the results of the CCK8 were consistent with the mitochondrial results: co-treatment with ATP and Cd significantly alleviated the decrease in Cd-induced cell activity (Fig. 9I, J). Compared with the Cd alone group, the ATP + Cd group showed alleviation of Cd-induced lipid accumulation (Fig. 9K–O).

### Rho-T1 restores liver mitochondrial transfer efficiency and energy metabolism

**Fig. 10:**
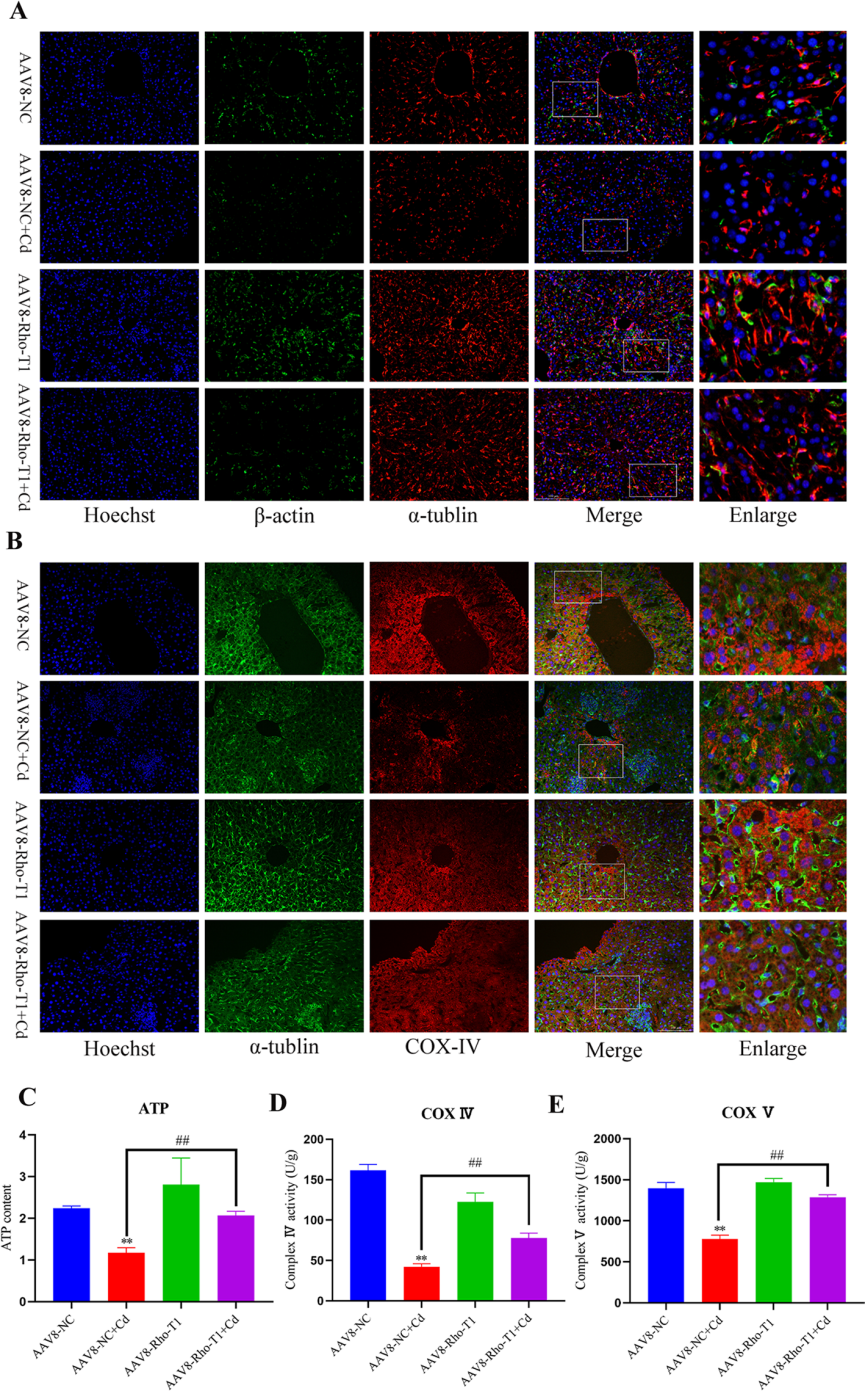
Rho-T1 restores liver mitochondrial transfer efficiency and energy metabolism. Co-staining of α-tubulin and β-actin to observe the effect of Cd on mouse liver tunnel nanotubes (**A**). Scale bar = 100 μm. Co-staining of α-tubulin and COX IV to observe the effect of Cd on intercellular mitochondrial transfer in the mouse liver (**B**). Scale bar = 100 μm. ATP contents were examined (**C**). The enzyme activities of the mitochondrial respiratory chain IV (**D**) and V (**E**) were examined. Results are shown as the mean ± SD (*n* = 3). Compared with the control group, *P < 0.05, **P < 0.01. Compared with the Cd group, ^#^*P* < 0.05, ^##^*P* < 0.01

To verify the prevalence of mitochondrial transfer, we examined the mitochondrial transfer efficiency in liver. We first observed the microtubules and F-actin, and the immunofluorescence results are shown in Fig. 10A. Cd destroyed the structure of the microtubules, which accumulates around the nucleus, and interestingly, after Cd treatment, the fluorescence intensity of F-actin also decreased to a certain extent. The Rho-T1 overexpression in the liver alleviated the changes of Cd to the structure of microtubules and F-actin. Subsequently, COX-IV. and α-tubulin were further co-stained, and we found that Cd reduced the fluorescence intensity and mitochondrial transfer efficiency of COX-IV in the liver, and overexpression of Rho-T1 alleviated the decrease in Cd-induced mitochondrial transfer efficiency (Fig. 10B), and we also tested the mitochondrial respiratory chain enzyme activity and ATP content, and the results showed that overexpression of Rho-T1 resisted the Cd-induced changes in enzyme activity and ATP content reduction (Fig. 10C–E).

### Rho-T1-mediated resistance to cadmium hepatotoxicity and lipid accumulation

**Fig. 11:**
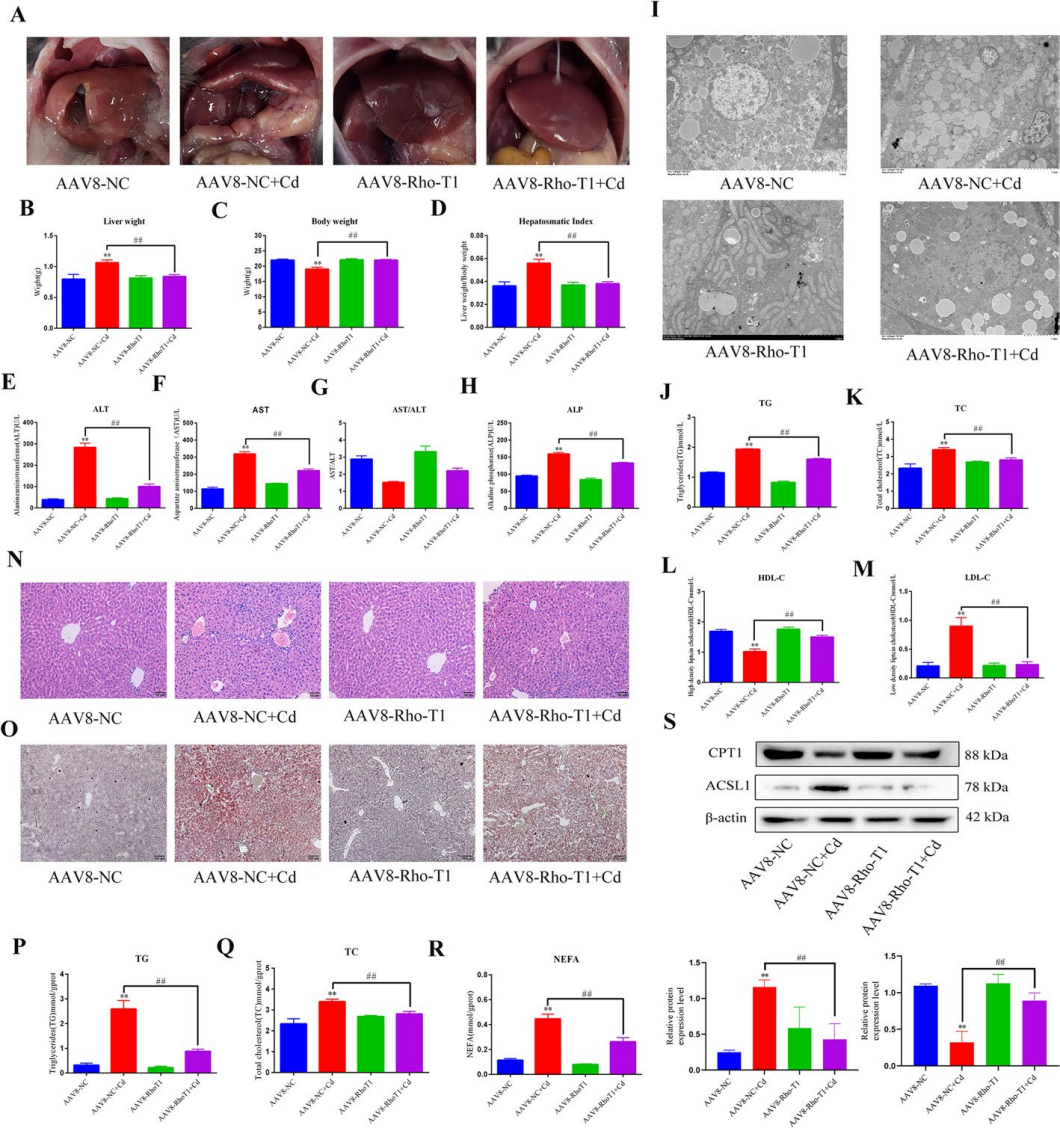
Rho-T1 resistance to cadmium hepatotoxicity and lipid accumulation. Morphological observation of the liver (**A**). Liver weights (**B**), Body weights (**C**), and Liver coefficient (**D**) in mice treated with Cd (1 mg/kg). Liver function was assessed according to serum levels of ALT (E), AST (F), AST/ALT (**G**), and ALP (**H**). Transmission electron microscopy to observe lipid droplets (**I**). Serum biochemistry to measure the levels of TG (**J**), TC (**K**), HDL-C (**L**), and LDL-C (**M**). Liver histopathology observed using HE staining (**N**). Scale bar = 50 μm. Oil red O staining to observe the content of neutral fat in tissues (**O**). Scale bar = 100 μm. TG, TC, and NEFA levels in the liver were measured (**P**–**R**). Levels of CPT1and ACSL1 were determined using western blotting (**S**). Results are shown as the mean ± SD (*n* = 3). Compared with the control group, *P < 0.05, **P < 0.01. Compared with the Cd group, ^#^*P* < 0.05, ^##^*P* < 0.01

To explore whether Rho-T1-mediated mitochondrial transfer can resist the hepatotoxicity of Cd, we first observed the morphological changes of the liver. The liver tissue of the Cd group had blunt and rounded edges and milky white plaque-like structures (Fig. 11A). AAV8-Rho-T1 + Cd alleviated the Cd-induced changes to liver morphology, body weight, liver weight, and liver coefficients (Fig. 11B–D). Alanine transaminase (ALT), aspartate transaminase (AST), and alkaline phosphatase (ALP) are important indicators of liver function, and the results showed that AAV8-RHOT1 reduces the amount ALT, AST, and ALP in the blood (Fig. 11E–H). We also measured blood levels of TG, TC, high-density lipoprotein-cholesterol (HDL-C), and low-density lipoprotein-cholesterol (LDL-C). The results showed that Cd increased the levels of TG, TC, and LDL-C in the blood, and decreased the levels of HDL-C. Compared with the Cd alone group, the Cd-induced changes to the blood parameters were partially restored in the AAV8-Rho-T1 + Cd group (Fig. 11J–M). Liver section HE staining, oil red O staining, and transmission electron microscopy (TEM) were used to visually observe the accumulation of liver lipid droplets. Cd significantly increased the intensity of oil red O staining, especially around the liver sinus, and AAV8-Rho-T1 + Cd reduced the intensity of oil red O staining (Fig. 11N, O). TEM showed that Cd reduced the size of the lipid droplets, but significantly increased the number of lipid droplets. The number of lipid droplets in the AAV8-Rho-T1 + Cd group decreased relative to that in the Cd group; however, there was no significant change in the size of the lipid droplets (Fig. 11I). TG, TC and free fatty acids (NEFA) in liver tissue were also measured, and the results were consistent with the blood levels (Fig. 11P–R). Finally, we examined CPT1 and ACSL1 levels in the liver, which showed that Cd decreased CPT1 levels and increased ACSL1 levels, and AAV8-Rho-T1 mitigated these Cd-induced protein changes (Fig. 11S).

## Discussion

The body is made up of countless cells, however, these cells do not exist alone and can be connected to other cells in various ways. Recent research revealed a new way of intercellular communication comprising tunneling nanotubes (TNTs). The composition, formation, and, mechanism of TNTs have become a research focus. F-actin was the first component of TNTs to be identified, which fills the interior of the tunnel. Microtubules are also components of the TNT structure [18]. However, there are differences in the ratio of F-actin and microtubules in different cell models [19, 20]. In this study, we found that there are multiple composition types in the hepatocyte model. One comprises nanotubes in which F-actin is the main component (up to 100%), and the other comprises nanotubes whose composition is dominated by microtubules. Interestingly, after Cd treatment, the microtubule-dominated nanotubes began to decrease, in a Cd concentration-dependent manner. The nanotubes also became thinner, and these change in nanotubes morphology also led to a change in function. Previous research focused on the regulation of actin filament networks, in which the activities of the small Rho GTPases Rac1, cdc42, and myosin X promote the formation of TNTs, and oxidative stress can also induce the formation of TNT through the PI3K pathway [19, 21, 22]. Interestingly, in the hepatocyte model, inhibition of actin polymerization did not inhibit the formation of TNTs, but promoted the formation of microtubule-dependent nanotubes. Considering the effects of Cd on tunneling TNTs, we speculated that microtubules are an important target of Cd. Indeed, after Noc inhibited the polymerization of microtubules, we found that the number of microtubule-dependent TNTs was reduced. The GSK-3β-Tau pathway regulates the stability of microtubules, and after Cd treatment, the stability of microtubules was reduced and the formation of microtubule-dependent TNTs was inhibited.

There are two main mechanisms of TNT formation, one depends on cell movement, in which two adjacent cells move in opposite directions to form a TNT, and the other originates from membrane protrusion, involving fusion of membrane protrusions between two cells or fusion of donor cell membrane protrusions with recipient cell membranes [23, 24]. In this study, we found that mitochondrial transfer originates from two adjacent cells, and as the cells separate, the nanotubes gradually form and function. After Cd treatment, the occurrence of mitochondrial transfer was inhibited. Whether mitochondrial transfer is directional has also been explored by researchers. In most stem cell models, mitochondria are shown to be transferred from stem cells to damaged cells [25], and some studies have shown that cancer cells can satisfy their own metabolic needs by acquiring mitochondria from normal cells [26]. Therefore, we explored whether mitochondrial transfer has directivity, and found that mitochondrial transfer is ubiquitous and bidirectional between liver cells. Interestingly, Cd treatment inhibited the transfer of mitochondria from normal cells to damaged cells, but promoted the transfer of mitochondria from damaged cells to normal cells, which might be similar to the release of mitochondria by damaged cells [27], Unfortunately, we have not yet determined the state of mitochondria during transfer, which will be addressed in a future study.

The movement of mitochondria is dependent on microtubules and motor proteins, and the movement of mitochondria from the perinuclear area to the cell membrane direction is defined as positive transport, whereas negative transport describes their movement in the reverse direction [28]. Studies have shown that Rho-T1 interacts with KIF5B to jointly drive the positive transport of mitochondria [29], with Rho-T1 being indispensable for mitochondrial transfer [30]. In this study, we found that Cd reduced Rho-T1 expression and attenuated its interaction with KIF5B. Overexpression of *RHOT1* and *KIF5B* reversed Cd inhibition of mitochondrial transfer. Therefore, we further speculate that negative mitochondrial transport also plays a role in cadmium inhibition of mitochondrial transport. Dynein is the motor protein mainly responsible for the negative transport of mitochondria, and Dynein and Dynactin form complexes that jointly drive the negative transport of mitochondria. However, we found that DYNLL1 levels were reduced after Cd treatment, suggesting that there might be an as-yet-undiscovered mechanism driving negative mitochondrial transport after Cd treatment. P62 is a highly conserved and versatile protein with numerous binding domains that can interact with a variety of proteins, participating in various cellular processes, such as autophagy, apoptosis, and inflammation [31–34]. P62 not only promotes the process of mitophagy as a ubiquitinated substrate, but also promotes the aggregation of mitochondria [35]. A previous study showed that Cd significantly increases P62 expression and is considered an important marker of Cd-induced blockade of autophagy flow [36]. However, this study showed that the elevation of P62 is not only the result of autophagic flow blockade, but also is required for mitochondrial aggregation. P62 can also regulate the remodeling of F-actin by interacting with HDAC6, which promotes the perinuclear aggregation of F-actin [37]. In this study, we found that with increasing Cd concentration, P62 fluorescent puncta increased and even showed a chain-like structure, which was co-localized with microtubules. We also found that P62 can interact with Dynaction1, which led us to propose the concept of microtubule-Dynaction1-P62 mitochondrial complexes. After silencing P62 expression, Cd-induced mitochondrial perinuclear aggregation and mitochondrial transfer inhibition were alleviated.

The role of mitochondrial transfer in different models and its significance have been the focus of our research, and we found that fat cells can transfer mitochondria to macrophages to regulate lipid metabolism and alleviate obesity [38]. In addition, platelets promote wound healing through mitochondrial transfer [39]. Hematopoietic stem cells transfer mitochondria to the stroma to promote bone marrow regeneration [20], and mitochondrial transfer has also been observed in bone cells and nerve cells [40, 41]. However, the presence and role of intercellular mitochondrial transfer in hepatocytes has rarely been reported. In this study, we observed mitochondrial transfer between hepatocytes. However, the role of mitochondrial transfer in Cd-induced hepatotoxicity is unclear. NAFLD has emerged as one of the important metabolic diseases in recent years, and its main feature is the accumulation of lipids [42]. Mitochondria are the main sites of lipid metabolism. Intravenous delivery of mitochondria might relieve NAFLD [43]. Mitochondria are also important targets for Cd toxicity; however, the effect of Cd inhibition of intercellular mitochondrial transfer on NAFLD is unclear. In this study, we found that Cd inhibits mitochondrial transfer and promotes NAFLD, and when Cd-induced inhibition of mitochondrial transfer was alleviated, the accumulation of lipids in cells was reduced. When exogenous mitochondria were co-cultured with damaged hepatocytes, lipid accumulation in the damaged cells was also relieved. This indicates that mitochondrial transfer protects against Cd-induced liver damage. The clinical application of mitochondrial transfer is mainly reflected in stem cell therapy. Stem cells can relieve lung damage and nerve damage via mitochondrial transfer [44, 45]. There is a lack of effective treatment for Cd poisoning, and thus mitochondrial transfer might represent a new idea for the treatment of Cd damage.

## Conclusion

In this study we reported mitochondrial transfer of hepatic parenchymal cells through microtubule-dependent TNTs. Cd disrupted microtubules and reduced the formation and function of TNTs. Cd disrupted KIF5B-Rho-T1-mediated positive mitochondrial transport and enhanced P62-Dynactin1-mediated negative mitochondrial transport, thereby reducing intercellular mitochondrial transfer. A decrease in mitochondrial transfer promotes the development of NAFLD.

### Supplementary Information

**Additional file 1: Figure S1.** AML12 cells were treated with 5 μM Cd for 3 (A)or 6(B) h. One group of CFDA-SE prestained cells was co-cultured with another population of Mito tracker-Red prestained cells for Flow cytometry to detect intercellular mitochondrial transfer efficiency. **Figure S2.** AML12 cells were treated with Cytd and Cd are processed separately or in combination for 6h, Confocal microscopy was used to observe colocalization of phalloidin and Tubulin-tracker-Red (A). Scale bar = 25 μm. Confocal microscopy was used to observe colocalization of phalloidin and TOMM20 (B). Scale bar = 25 μm. Scanning electron microscopy was used to observe intercellular tunneling nanotube structures (C). Scale bar = 50 μm. Oil red O (D) was used to observe intracellular lipid droplets. Scale bar = 100 μm. **Figure S3.** AML12 cells were treated with Noc and Cd are processed separately or in combination for 6h, Confocal microscopy was used to observe colocalization of TOMM20 and Tubulin-tracker-Red (A). Scale bar = 25 μm. **Figure S4.** AML12 cells were treated with Noc and Cd are processed separately or in combination for 6h, Confocal microscopy was used to observe colocalization of phalloidin and Mito-tracker-Red (A). Scale bar = 25 μm.

**Additional file 2.** Movie S1.

**Additional file 3.** Movie S2.

## Data Availability

The datasets used and/or analysed during the current study are available from the corresponding author on reasonable request.

## Abbreviations

Cd: Cadmium
NAFLD: Nonalcoholic fatty liver disease
TNT: Tunneling nanotubes
AML12: Alpha mouse liver 12
CFDA-SE: Carboxyfluorescein diacetate succinimidyl ester
TOMM20: Translocase of outer mitochondrial membrane 20
CPT1: Carnitine palmitoyltransferase 1
ACSL1: Acyl-CoA synthetase long chain family member 1
GSK-3β: Glycogen synthase kinase 3 beta
COX IV: Cytochrome C oxidase subunit 4
P62: Sequestosome 1
KIF5B: Kinesin family member 5B
Rho-T1: Mitochondrial Rho GTPase 1
DYNLL1: Dynein light chain LC8-type 1
TG: Triglyceride
TC: Total cholesterol
Noc: Nocodazole
Cytd: Cytochalasin D
CCK8: Cell Counting Kit-8
siRNAs: Small interfering RNAs
ITS-A: Insulin-Transferrin-Selenium-Sodium Pyruvate
FBS: Fetal bovine serum
PBS: Phosphate-buffered saline
OTC: Optimal cutting temperature
RIPA: Radioimmunoprecipitation assay
BCA: Bicinchoninic acid
TBST: Tris Buffered Saline with Tween^®^ 20
SEM: Scanning electron microscopy
TEM: Transmission electron microscopy

## References

[1] Samuel VT, Shulman GI (2018). Nonalcoholic fatty liver disease as a nexus of metabolic and hepatic diseases. Cell Metab.

[2] Day CP, James OF (1998). Steatohepatitis: a tale of two “hits”?. Gastroenterology.

[3] Nguyen HD, Kim MS (2022). Cadmium, lead, and mercury mixtures interact with non-alcoholic fatty liver diseases. Environ Pollut.

[4] Ma Y, Ran D, Cao Y, Zhao H, Song R, Zou H (2021). The effect of P2X7 on cadmium-induced osteoporosis in mice. J Hazard Mater.

[5] Rui L (2014). Energy metabolism in the liver. Compr Physiol.

[6] Ng MYW, Wai T, Simonsen A (2021). Quality control of the mitochondrion. Dev Cell.

[7] Glancy B, Kim Y, Katti P, Willingham TB (2020). The functional impact of mitochondrial structure across subcellular scales. Front Physiol.

[8] Suh J, Kim NK, Shim W, Lee SH, Kim HJ, Moon E (2023). Mitochondrial fragmentation and donut formation enhance mitochondrial secretion to promote osteogenesis. Cell Metab.

[9] Melkov A, Abdu U (2018). Regulation of long-distance transport of mitochondria along microtubules. Cell Mol Life Sci.

[10] Rowland AA, Voeltz GK (2012). Endoplasmic reticulum-mitochondria contacts: function of the junction. Nat Rev Mol Cell Biol.

[11] Wong YC, Ysselstein D, Krainc D (2018). Mitochondria-lysosome contacts regulate mitochondrial fission via RAB7 GTP hydrolysis. Nature.

[12] Shanmughapriya S, Langford D, Natarajaseenivasan K (2020). Inter and Intracellular mitochondrial trafficking in health and disease. Ageing Res Rev.

[13] Cordero Cervantes D, Zurzolo C (2021). Peering into tunneling nanotubes-the path forward. EMBO J.

[14] Kim BW, Lee JS, Ko YG (2019). Mycoplasma exploits mammalian tunneling nanotubes for cell-to-cell dissemination. BMB Rep.

[15] Panasiuk M, Rychlowski M, Derewonko N, Bienkowska-Szewczyk K. Tunneling nanotubes as a novel route of cell-to-cell spread of herpesviruses. J Virol. 2018;92(10). 10.1128/JVI.00090-18 PMC5923070 29491165

[16] Velarde F, Ezquerra S, Delbruyere X, Caicedo A, Hidalgo Y, Khoury M (2022). Mesenchymal stem cell-mediated transfer of mitochondria: mechanisms and functional impact. Cell Mol Life Sci.

[17] Alshehri AS, El-Kott AF, El-Kenawy AE, Khalifa HS, AlRamlawy AM (2021). Cadmium chloride induces non-alcoholic fatty liver disease in rats by stimulating miR-34a/SIRT1/FXR/p53 axis. Sci Total Environ.

[18] Ljubojevic N, Henderson JM, Zurzolo C (2021). The ways of actin: why tunneling nanotubes are unique cell protrusions. Trends Cell Biol.

[19] Keller KE (2020). Tunneling nanotubes and actin cytoskeleton dynamics in glaucoma. Neural Regen Res.

[20] Burt R, Dey A, Aref S, Aguiar M, Akarca A, Bailey K (2019). Activated stromal cells transfer mitochondria to rescue acute lymphoblastic leukemia cells from oxidative stress. Blood.

[21] Lappalainen P, Kotila T, Jegou A, Romet-Lemonne G (2022). Biochemical and mechanical regulation of actin dynamics. Nat Rev Mol Cell Biol.

[22] Zhang Y (2011). Tunneling-nanotube: a new way of cell-cell communication. Commun Integr Biol.

[23] Davis DM, Sowinski S (2008). Membrane nanotubes: dynamic long-distance connections between animal cells. Nat Rev Mol Cell Biol.

[24] Zampieri LX, Silva-Almeida C, Rondeau JD, Sonveaux P (2021). Mitochondrial transfer in cancer: a comprehensive review. Int J Mol Sci.

[25] Wang J, Li H, Yao Y, Zhao T, Chen YY, Shen YL (2018). Stem cell-derived mitochondria transplantation: a novel strategy and the challenges for the treatment of tissue injury. Stem Cell Res Ther.

[26] Saha T, Dash C, Jayabalan R, Khiste S, Kulkarni A, Kurmi K (2022). Intercellular nanotubes mediate mitochondrial trafficking between cancer and immune cells. Nat Nanotechnol.

[27] Rosina M, Ceci V, Turchi R, Chuan L, Borcherding N, Sciarretta F (2022). Ejection of damaged mitochondria and their removal by macrophages ensure efficient thermogenesis in brown adipose tissue. Cell Metab.

[28] Kruppa AJ, Buss F. Motor proteins at the mitochondria-cytoskeleton interface. J Cell Sci. 2021;134(7). 10.1242/jcs.226084 PMC8077471 33912943

[29] Las G, Shirihai OS (2014). Miro1: new wheels for transferring mitochondria. EMBO J.

[30] Ahmad T, Mukherjee S, Pattnaik B, Kumar M, Singh S, Kumar M (2014). Miro1 regulates intercellular mitochondrial transport & enhances mesenchymal stem cell rescue efficacy. EMBO J.

[31] Deng Z, Lim J, Wang Q, Purtell K, Wu S, Palomo GM (2020). ALS-FTLD-linked mutations of SQSTM1/p62 disrupt selective autophagy and NFE2L2/NRF2 anti-oxidative stress pathway. Autophagy.

[32] Moscat J, Diaz-Meco MT (2009). p62 at the crossroads of autophagy, apoptosis, and cancer. Cell.

[33] Zhou L, He X, Wang L, Wei P, Cai Z, Zhang S (2022). Palmitoylation restricts SQSTM1/p62-mediated autophagic degradation of NOD2 to modulate inflammation. Cell Death Differ.

[34] Liu WJ, Ye L, Huang WF, Guo LJ, Xu ZG, Wu HL, et al. p62 links the autophagy pathway and the ubiqutin-proteasome system upon ubiquitinated protein degradation. Cell Mol Biol Lett. 2016;21. 10.1186/s11658-016-0031-z PMC5415757 28536631

[35] Narendra D, Kane LA, Hauser DN, Fearnley IM, Youle RJ (2010). p62/SQSTM1 is required for Parkin-induced mitochondrial clustering but not mitophagy; VDAC1 is dispensable for both. Autophagy.

[36] Sun J, Yu F, Wang T, Bian J, Liu Z, Zou H (2022). The role of DRP1- PINK1-Parkin-mediated mitophagy in early cadmium-induced liver damage. Toxicology.

[37] Yan J, Seibenhener ML, Calderilla-Barbosa L, Diaz-Meco MT, Moscat J, Jiang J (2013). SQSTM1/p62 interacts with HDAC6 and regulates deacetylase activity. PLoS ONE.

[38] Brestoff JR, Wilen CB, Moley JR, Li Y, Zou W, Malvin NP (2021). Intercellular mitochondria transfer to macrophages regulates white adipose tissue homeostasis and is impaired in obesity. Cell Metab.

[39] Levoux J, Prola A, Lafuste P, Gervais M, Chevallier N, Koumaiha Z (2021). Platelets facilitate the wound-healing capability of mesenchymal stem cells by mitochondrial transfer and metabolic reprogramming. Cell Metab.

[40] Gao J, Qin A, Liu D, Ruan R, Wang Q, Yuan J (2019). Endoplasmic reticulum mediates mitochondrial transfer within the osteocyte dendritic network. Sci Adv.

[41] Vargas JY, Loria F, Wu YJ, Cordova G, Nonaka T, Bellow S (2019). The Wnt/Ca(2+) pathway is involved in interneuronal communication mediated by tunneling nanotubes. EMBO J.

[42] Byrne CD, Targher G (2015). NAFLD: a multisystem disease. J Hepatol.

[43] Fu A, Shi X, Zhang H, Fu B (2017). Mitotherapy for fatty liver by intravenous administration of exogenous mitochondria in male mice. Front Pharmacol.

[44] Islam MN, Das SR, Emin MT, Wei M, Sun L, Westphalen K (2012). Mitochondrial transfer from bone-marrow-derived stromal cells to pulmonary alveoli protects against acute lung injury. Nat Med.

[45] Boukelmoune N, Chiu GS, Kavelaars A, Heijnen CJ (2018). Mitochondrial transfer from mesenchymal stem cells to neural stem cells protects against the neurotoxic effects of cisplatin. Acta Neuropathol Commun.

