# Supplementary material for: Cadmium promotes nonalcoholic fatty liver disease by inhibiting intercellular mitochondrial transfer
Source: Cell Mol Biol Lett. 2023 Oct 27;28:87. doi: 10.1186/s11658-023-00498-x (PMC10604759; doi:10.1186/s11658-023-00498-x)
Supplement: Supplementary file 1 — Additional file 1: Figure S1. AML12 cells were treated with 5 μM Cd for 3 (A)or 6(B) h. One group of CFDA-SE prestained cells was co-cultured with another population of Mito tracker-Red prestained cells for Flow cytometry to detect intercellular mitochondrial transfer efficiency. Figure S2. AML12 cells were treated with Cytd and Cd are processed separately or in combination for 6h, Confocal microscopy was used to observe colocalization of phalloidin and Tubulin-tracker-Red (A). Scale bar = 25 μm. Confocal microscopy was used to observe colocalization of phalloidin and TOMM20 (B). Scale bar = 25 μm. Scanning electron microscopy was used to observe intercellular tunneling nanotube structures (C). Scale bar = 50 μm. Oil red O (D) was used to observe intracellular lipid droplets. Scale bar = 100 μm. Figure S3. AML12 cells were treated with Noc and Cd are processed separately or in combination for 6h, Confocal microscopy was used to observe colocalization of TOMM20 and Tubulin-tracker-Red (A). Scale bar = 25 μm. Figure S4. AML12 cells were treated with Noc and Cd are processed separately or in combination for 6h, Confocal microscopy was used to observe colocalization of phalloidin and Mito-tracker-Red (A). Scale bar = 25 μm. [file 11658_2023_498_MOESM1_ESM.docx]

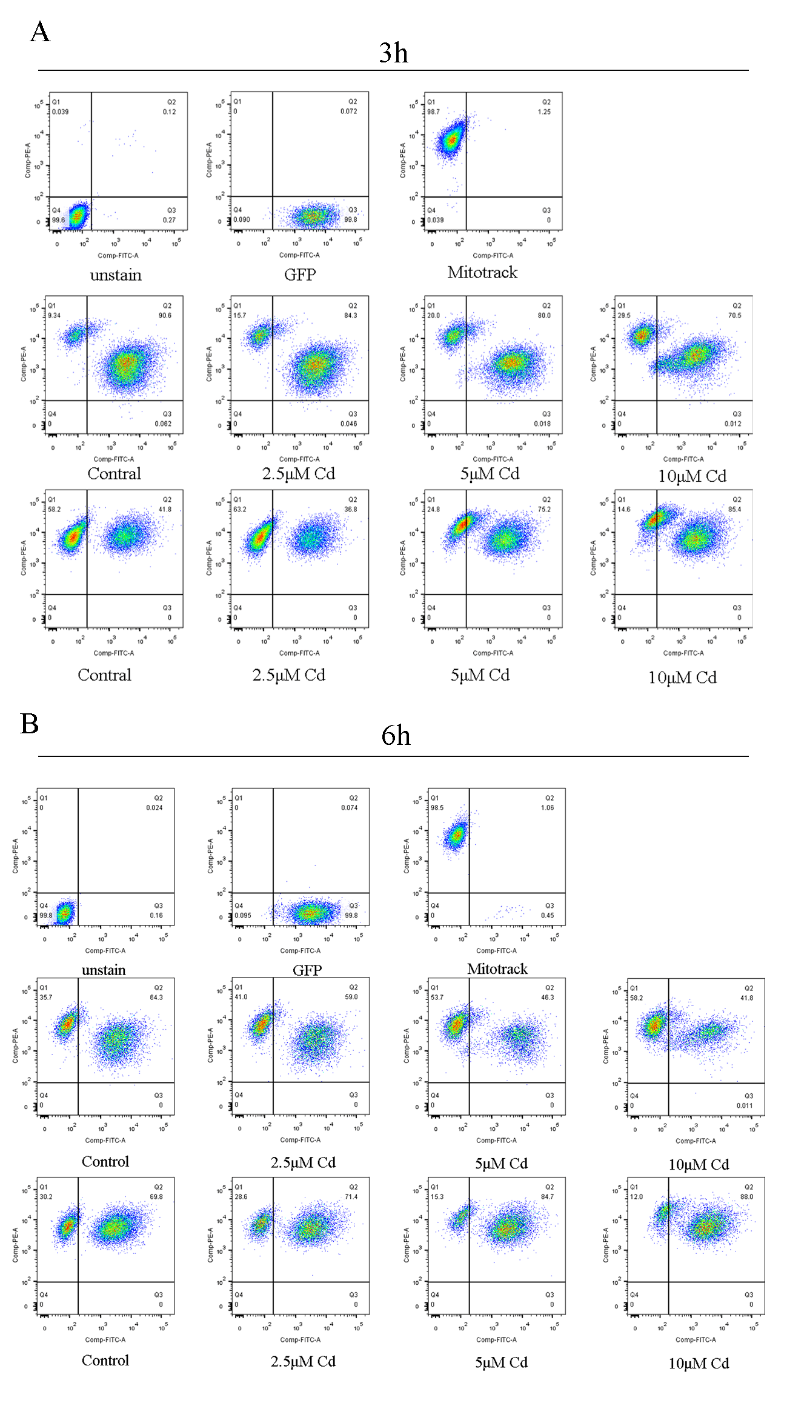


FigureS1: AML12 cells were treated with 5 μM Cd for 3 (A)or 6(B) h. One group of CFDA-SE prestained cells was co-cultured with another population of Mito‑tracker-Red prestained cells for Flow cytometry to detect intercellular mitochondrial transfer efficiency.


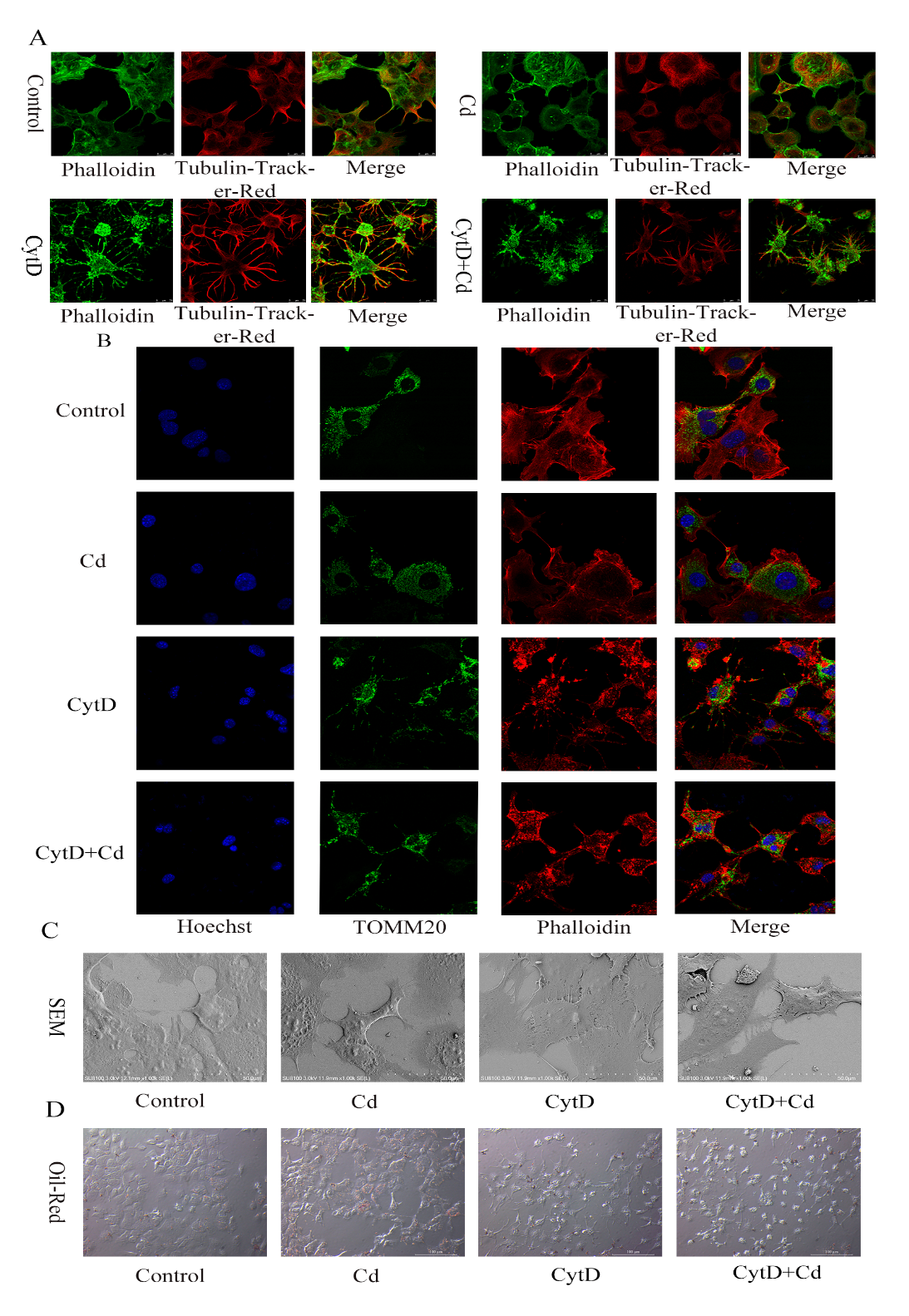


FigureS2: AML12 cells were treated with Cytd and Cd are processed separately or in combination for 6h, Confocal microscopy was used to observe colocalization of phalloidin and Tubulin-tracker-Red (A). Scale bar = 25 μm. Confocal microscopy was used to observe colocalization of phalloidin and TOMM20 (B). Scale bar = 25 μm. Scanning electron microscopy was used to observe intercellular tunneling nanotube structures (C). Scale bar = 50 μm. Oil red O (D) was used to observe intracellular lipid droplets. Scale bar = 100 μm.


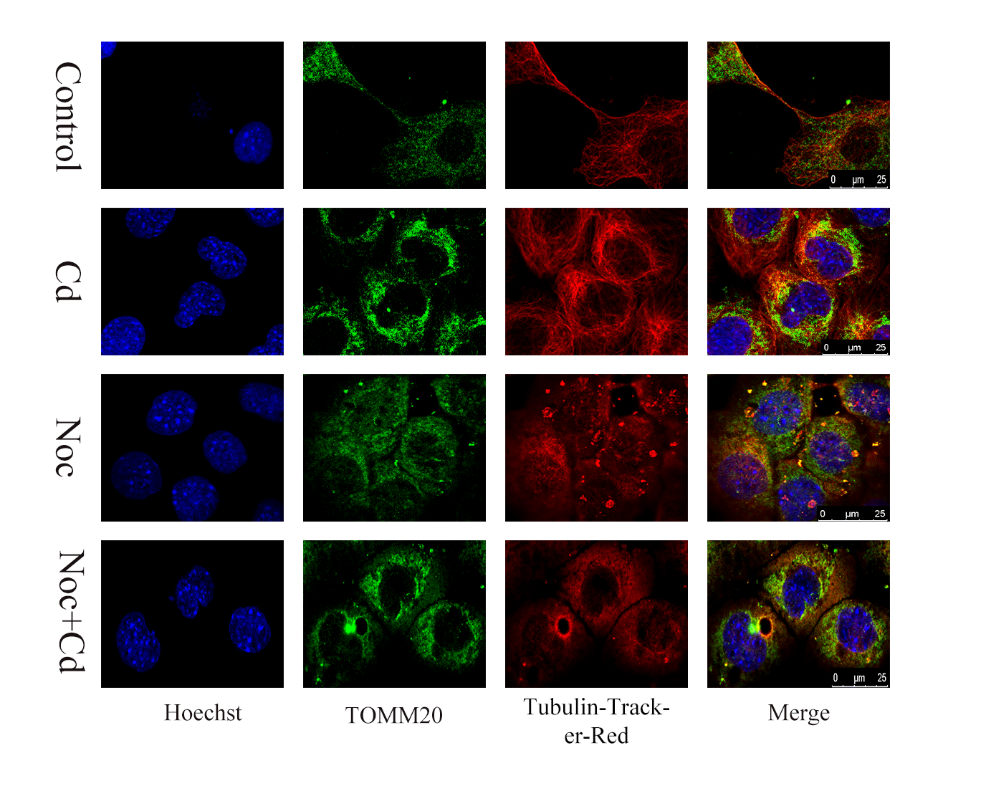


FigureS3: AML12 cells were treated with Noc and Cd are processed separately or in combination for 6h, Confocal microscopy was used to observe colocalization of TOMM20 and Tubulin-tracker-Red (A). Scale bar = 25 μm.


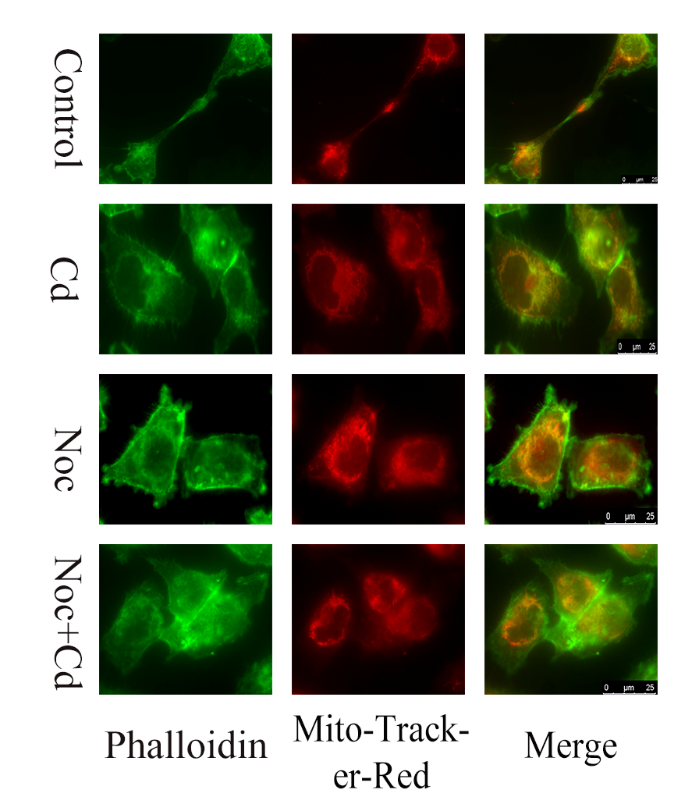


FigureS4: AML12 cells were treated with Noc and Cd are processed separately or in combination for 6h, Confocal microscopy was used to observe colocalization of phalloidin and Mito-tracker-Red (A). Scale bar = 25 μm.
